# Impact of Asialoglycoprotein Receptor and Mannose Receptor Deficiency on Murine Plasma N-glycome Profiles

**DOI:** 10.1016/j.mcpro.2023.100615

**Published:** 2023-07-04

**Authors:** M. Svecla, J. Nour, M.R. Bladergroen, S. Nicolardi, T. Zhang, G. Beretta, M. Wuhrer, G.D. Norata, D. Falck

**Affiliations:** 1 Department of Pharmacological and Biomolecular Sciences, Università degli Studi di Milano, Milan, Italy; 2 Center for Proteomics and Metabolomics, Leiden University Medical Center, Leiden, The Netherlands; 3 Department of Environmental Science and Policy, Università degli Studi di Milano, Milan, Italy; 4 Centro SISA per lo studio dell’Aterosclerosi, Ospedale Bassini, Cinisello Balsamo, Italy

**Keywords:** asialoglycoprotein receptor, mannose receptor, clearance, glycomics, proteomics

## Abstract

The asialoglycoprotein receptor (ASGPR) and the mannose receptor C-type 1 (MRC1) are well known for their selective recognition and clearance of circulating glycoproteins. Terminal galactose and *N*-Acetylgalactosamine are recognized by ASGPR, while terminal mannose, fucose, and *N*-Acetylglucosamine are recognized by MRC1. The effects of ASGPR and MRC1 deficiency on the N-glycosylation of individual circulating proteins have been studied. However, the impact on the homeostasis of the major plasma glycoproteins is debated and their glycosylation has not been mapped with high molecular resolution in this context. Therefore, we evaluated the total plasma N-glycome and plasma proteome of ASGR1 and MRC1 deficient mice. ASGPR deficiency resulted in an increase in O-acetylation of sialic acids accompanied by higher levels of apolipoprotein D, haptoglobin, and vitronectin. MRC1 deficiency decreased fucosylation without affecting the abundance of the major circulating glycoproteins. Our findings confirm that concentrations and N-glycosylation of the major plasma proteins are tightly controlled and further suggest that glycan-binding receptors have redundancy, allowing compensation for the loss of one major clearance receptor.

## Highlights

• Plasma N-glycome remains stable after removal of major glycoprotein binding receptors.
• ASGPR deficiency increased O-acetylation instead of terminal galactosylation.
• MRC1 deficiency had no effect on oligomannose glycans but decreased fucosylation.

## Graphical Abstract

**Figure:**
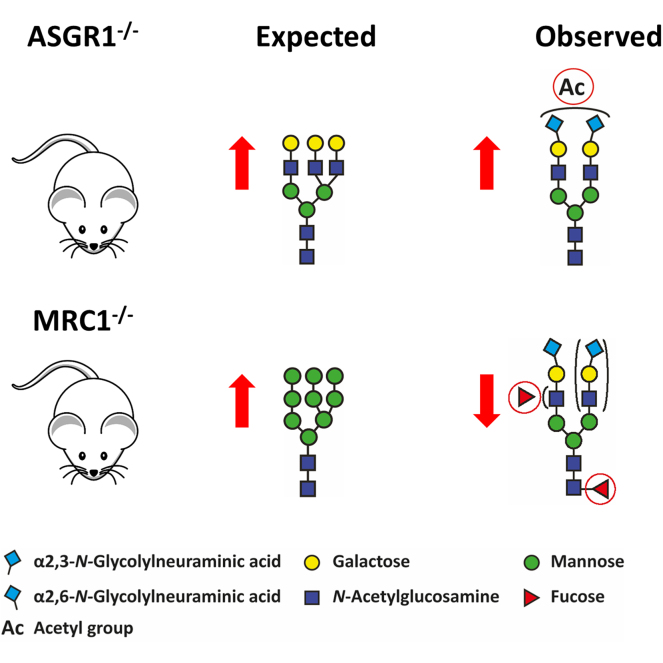

## In Brief

C-type lectins on liver cells clear glycoproteins from circulation. Investigating controversial aspects, we found very limited changes in the plasma N-glycome and proteome of receptor-deficient mice. For the asialoglycoprotein receptor, we excluded an accumulation of terminal galactose or α2,6-linked sialylation and instead observed an accumulation of sialic acid O-acetylation. For the mannose receptor, oligomannose-carrying glycoproteins were not increased, but instead, fucosylated glycoproteins were decreased in deficient mice. We conclude that the plasma glycoproteome is tightly regulated *via* compensatory mechanisms.

Glycan moieties are crucial when it comes to glycoprotein homeostasis and clearance from circulation. Modulating glycan patterns have been shown to change the concentration and the half-life of glycoproteins, and therefore their rate of clearance from the circulation (1). The affinity for glycan-binding proteins with strong motif preferences governs the selective recognition, internalization, and clearance of glycoproteins from circulation. C-type lectins (CTLs), which contain a Ca^2+^-dependent carbohydrate recognition domain, are the largest glycan-binding protein family that interacts with circulating glycoproteins (2). The asialoglycoprotein receptor (ASGPR) and the mannose receptor C-type 1 (MRC1) are the two CTLs most often reported to be involved in the selective clearance of circulating glycoproteins.

An important factor in the clearance of plasma glycoproteins is desialylation, the removal of sialic acid as a terminal monosaccharide by circulating sialidases (NEU1,3,4) (3). This exposes galactose (Gal) or *N*-acetylgalactosamine (GalNAc) as terminal glycan moieties which are recognized by ASGPR, also known as Ashwell-Morell receptor, initiating protein clearance (4, 5). Interestingly, only α2,3-sialylation abolishes ASGPR affinity while α2,6-sialylation does not (6). Furthermore, O-acetylation of sialic acids protects them from desialylation by reducing sialidase activity (7). Our information on these phenomena is derived from mouse models where plasma protein sialylation is predominantly *N*-glycolylneuraminic acid (8). In contrast, circulating glycoproteins carrying terminal mannose, fucose, or *N*-acetylglucosamine (GlcNAc) are recognized and cleared by MRC1 (9, 10). However, the affinity for fucose has only been described in the context of Lewis-type structures which feature antennary fucosylation (α1,2-, α1,3- or α1,4-linkage) distinct from core fucosylation (α1,6-linkage) (10). ASGPR is composed of two subunits, the major (ASGR1) and the minor (ASGR2), where only the major subunit is required for the expression of a functional receptor (11). On the other hand, MRC1 (known also as CD206) presents different ligand-binding domains. The extracellular region displays: a cysteine-rich domain able to bind sulfated carbohydrates, a collagen-binding fibronectin type II (FNII) domain, and 8 C-type lectin domains, of which only the fourth is functional and recognizes mannose, fucose, or GlcNAc as terminal glycan moiety in circulating glycoproteins (12, 13, 14).

Both receptors are highly expressed in the liver—ASGPR primarily on the surface of hepatocytes, MRC1 on liver sinusoidal endothelial cells, and on the surface of macrophages (15). Regarding these ligands, ASGPR has been shown to be involved in the clearance of circulating IgA (16, 17), low-density lipoproteins (LDL) (18, 19), chylomicron remnants (20), cellular fibronectin (21), and platelets (22). Additionally, in mouse models, the lack of ST3GAL4 is associated with platelet deficiency. CMP-*N*-acetylneuraminate-β-galactosamide-α-2,3-sialyltransferase 4 (ST3GAL4) catalyzes the α2,3-sialylation of the terminal Gal moiety that prevents the recognition and clearance of circulating glycoproteins by ASGPR. However, the shortened half-life of platelets was restored to normal by the additional loss of ASGR1 (22). This suggests that linkage-specific sialylation by ST3GAL4 is required to maintain the normal half-life of the platelets. Interestingly, although the role of ASGPR in clearing individual glycoproteins with terminal galactose is well established, there is no consensus on whether ASGPR deficiency has a major impact on circulating glycoproteins (5, 22). Meanwhile, MRC1 has been shown to be involved in the clearance of lysosomal enzymes (23), collagen fragments (13), and pituitary hormones (24, 25). Its role in the preferential clearance of oligomannosidic glycoforms of therapeutic monoclonal antibodies has also been suggested (26). Interestingly, MRC1 is known also for pathogen recognition and clearance including, *C. albicans* (27), *Pneumocystis carinii* (28), and *Leishmania donovani* (29).

Consequently, ASGPR and MRC1 are involved in a wide range of biological processes. For example, deletion of these receptors leads to increased levels of luteinizing hormone and testosterone in mice, accompanied by impaired reproduction (15). Furthermore, human data in a loss-of-function ASGR1 variant are associated with lower plasma lipid levels and a 34% reduction in coronary artery disease (CAD) (30). In contrast, increased plasma levels of soluble mannose receptors have been linked to several inflammatory diseases in humans and mice (31, 32, 33). Furthermore, increased plasma mannose concentration is thought to be a promising biomarker for CAD risk (34).

Due to the glycan specificity of those receptors, alterations in branching and terminal residues can have a strong impact on the glycoprotein circulator half-life. To investigate *in vivo* the impact of ASGPR and MRC1 on the N-glycosylation of circulating proteins, we profiled total plasma N-glycome (TPNG) and performed shotgun plasma proteomics in ASGR1 and MRC1 deficient mice. TPNG is a collection of N-glycans released from the major plasma proteins that are frequently used in biomedical and clinical investigations (35, 36).

## Experimental Procedures

### Animals

ASGR1^−/−^ mice (B6.129S4-Asgr1tm1Sau/SaubJxm) were purchased from Jackson Laboratory while MRC1^−/−^ mice (B6.129P2-Mrc1tm1Mnz/J) were in-house generated. The wild-type (WT) controls for all experiments were the offspring backcrossed ASGR1^−/−^ or MRC1^−/−^ littermates. The mice were kept under a controlled light/dark cycle (12 h of light/12 h of dark) and temperature-controlled conditions (21 °C). Starting at 8 weeks of age, for 20 weeks, mice were fed on a high-fat diet (HFD- 45% Kcal from fat, Research Diets, Inc Cat#D12451), and water was provided *ad libitum*. The procedures were performed conforming to the guidelines from the 2010/63/EU directive of the European Parliament on the protection of animals used for scientific purposes and were approved by the Ethical Committee of the University of Milan and the Italian Ministry of Health (Progetto di Ricerca 91/2020, 929/2020).

### Samples

Blood samples were collected by intracardiac puncture with disodium EDTA as an anticoagulant, and plasma was separated by centrifugation at 7800*g* for 10 min. As a technical control, a pool (n = 7) of WT mouse plasma was used.

### Experimental Design and Statistical Rational

The TPNG profiles were conducted in ASGR1^−/−^ (n = 12) *versus* WT (n = 9), and MRC1^−/−^ (n = 10) *versus* WT (n = 8). While the proteomics profile was carried out in ASGR1^−/−^ (n = 4) *versus* WT (n = 4), and MRC1^−/−^ (n = 4) *versus* WT (n = 4). To reduce cofounders, mice were generated as littermates and kept in the same room, and for reproducibility in our study are included three cohorts of animals from each genotype. The data were normally distributed, and thus a parametric *t* test is applied. For multiple comparisons of the glycosylation traits, the Bonferroni-Dunn method with a *p* < 0.05 was applied (37, 38).

### N-Glycan Release and Linkage-Specific Sialic Acid Derivatization for MALDI-FT-ICR Analysis

The N-glycan release from plasma was performed with peptide-N-glycosidase F (PNGaseF, Roche). Firstly, for denaturation, 6 μl of plasma were added to 12 μl 2 % SDS and incubated for 10 min at 60 °C. After incubation 12.6 μl was added as a release mixture (6 μl 4 % NP40, 6 μl 5 × PBS and 0.6 μl PNGase F), and the samples were incubated overnight at 37 °C. Sialic acid derivatization was performed as previously described (39). Two μl of released glycans were added to 40 μl of ethyl esterification reagent (0.25 M 1-ethyl-3-(3-dimethylamino)propyl)carbodiimide with 0.25 M 1-hydroxybenzotriazole in ethanol), after which the mixture was incubated for 1 h at 37 °C. This introduced a mass difference between α2,3-linked sialic acids, losing water through lactonization, and α2,6-linked sialic acids, gaining C_2_H_4_ through esterification with ethanol (40). Subsequently, 40 μl of acetonitrile was added and after 10 min of incubation, the purification was started. In-house assembled microtips used for cotton hydrophilic interaction liquid chromatography (HILIC), microtip purification were prepared as follows: 3 mm cotton thread (approximately 180 μg, Pipoos) were placed into a 50 μl tip (clear CO-RE tip without filter, Hamilton) by using tweezers. Then, a porous polypropylene frit (DPX Technologies) was placed 18 mm above the tip opening. The cotton HILIC tips were three times pre-wetted with 40 μl of MQ water and then conditioned with three times 40 μl of 85% ACN. Subsequently, the sample was loaded by pipetting the ethyl-esterified sample 20 times up and down (40 μl per time). The HILIC tips were washed three times with 40 μl of 85% ACN containing 1% trifluoroacetic acid (TFA), and three times with 40 μl of 85% ACN. The purified N-glycans were eluted in 20 μl of MQ water by pipetting five times up and down (pipet set at 15 μl). Next, 10 μl of purified sample was premixed with 5 μl of sDHB matrix (5 mg/ml in 99% ACN with 1 mM NaOH, Sigma-Aldrich) and 3 μl of the mixture was spotted onto a MALDI target plate (800/384 MTP AnchorChip, Bruker Daltonics).

### In-Solution Trypsin Digestion for Plasma Proteomics

For plasma proteomics (n = 4 each) were pooled and the concentration was quantified by NanoDrop A280 nm (Thermo Fisher). 20 μl of plasma was mixed with 40 μl of Ammonium bicarbonate solution 50 mM (final pH = 8.5). Proteins were reduced by incubation with 3 μl DTT 100 mM, for 30 min at 55 °C. Protein alkylation was then performed at room temperature, by incubating with 6 μl of iodoacetamide 150 mM, for 20 min in the dark. Trypsin digestion (enzyme to protein ratio 1:20), was performed overnight at 37 °C and stopped by acidification with trifluoroacetic acid (final percentage 1%).

### Matrix-Assisted Laser Desorption/Ionization Fourier Transform ion Cyclotron Resonance Mass Spectrometry Analysis of Released N-Glycans

Matrix-Assisted Laser Desorption/Ionization Fourier Transform ion Cyclotron Resonance Mass Spectrometry experiments were performed similarly to previous reports (41). Briefly, all MS experiments were performed on a Bruker 15 T solariX XR FT-ICR mass spectrometer equipped with a CombiSource and a ParaCell (Bruker Daltonics, Bremen, Germany). The FT-ICR MS system was controlled by the ftmsControl software and equipped with a Smartbeam-II Laser System (Bruker Daltonics) that operated at a frequency of 500 Hz. Every single spectrum was generated from 200 laser shots. The mass spectra were obtained from a single spot in the m/z range of 1000 to 5000. The data were acquired in serial mode and a single combined file was generated.

### LC-MS/MS Analysis of Plasma Proteome

An Ultimate 3000 nano-LC system (Thermo Fisher Scientific) connected to an Orbitrap Fusion Tribrid Mass spectrometer (Thermo Fisher Scientific) equipped with a nanoelectrospray ion source was used for plasma proteomics. Peptide mixtures were pre-concentrated into an Acclaim PepMap 100 to 100 μm × 2 cm C18 (Thermo Fisher Scientific) and separated on EASY-Spray column ES802A, 25 cm × 75 μm ID packed with Thermo Scientific Acclaim PepMap RSLC C18, 3 μm, 100 Å using mobile phase A (0.1 % formic acid in water) and mobile phase B (0.1% aqueous formic acid/acetonitrile (2:8)) with the following elution gradient: 4 to 28% for 90 min, 28 to 40% for 1 min, followed by 95% for a total runtime of 150 min, at a flow rate of 300 nl/min. The temperature of the column was set to 35 °C and the sample was injected four times. The injection volume was 3 μl for each sample. Two blanks were run between samples to prevent sample carryover. MS spectra were collected in positive ion mode over an m/z range of 375 to 1500 Da (resolution 120,000), automatic gain control (AGC) target 4 × 105, maximum injection time of 50 ms, operating in the data-dependent mode, cycle time 3 s between master scans. MS/MS spectra were collected in centroid mode. HCD was performed with collision energy set at 35 eV.

### Analysis of Released N-Glycans Using PGC Nano-LC-ESI-MS/MS

*N*-glycan alditols released from serum were prepared using a 96-well plate sample preparation method performed as previously described (42). In brief, 10 μL was applied to the hydrophobic Immobilon-P PVDF membrane in a 96-well plate format. Protein denaturation was achieved by applying 75 μl denaturation mix (72.5 μl 8 M GuHCl) and 2.5 μl 200 mM DTT) in each well, followed by shaking for 15 min and incubating at 60 °C in a moisture box for 30 min. Subsequently, the unbound material was removed by centrifugation. The N-glycan was released by adding peptide-N-glycosidase F (PNGase F) (2 U of enzyme diluted with water to 15 μl) to each well and incubated overnight at 37 °C. Released N-glycans were collected from the PVDF plate by centrifugation, and the glycosylamine versions of the released N-glycans were hydrolyzed by adding 20 μl of 100 mM ammonium acetate (pH 5), incubated at room temperature (RT) for 1 h, and dried in a SpeedVac concentrator 5301 (Eppendorf) at 35 °C. Collected N-glycans were then reduced and desalted followed by PGC cleanup using a 96-well plate-based protocol (42). Samples were dried in a SpeedVac concentrator directly in PCR plates and re-dissolved in 10 μl of water prior to porous graphitized carbon nano-liquid chromatography (PGC nano-LC-ESI-MS/MS) analysis. A home-packed PGC trap column (5 μm Hypercarb, 320 μm × 30 mm) and a home-packed PGC nano-column (3 μm Hypercarb 100 μm × 150 mm) connected to an amaZon ETD speed ion trap were used for the detection of N-glycans (Bruker Daltonics). Mobile phase B was 60% (v/v) acetonitrile/10 mM ABC, while mobile phase A contained 10 mM ABC. A multi-step gradient of B was used to achieve separation: 2 to 9% in 1 min, 9 to 49% in 80 min, and then a 10-min wash phase using 95% of B at a flow rate of 0.6 μl/min. The temperature of the column was maintained at 45 degrees. Ionization was achieved using the CaptiveSpray nanoBooster source (Bruker) with a capillary voltage of 1000 V applied, dry gas temperature of 280 °C at 5 L min^−1^, and nebulizer at 3-pound per square inch (psi). Isopropanol-enriched dopant nitrogen was used. MS spectra were acquired within a m/z range of 500 to 1850 for N-glycans, smart parameter setting (SPS) was set to *m*/z 1200; ion charge control (ICC) to 4 × 10^3^, and maximum acquisition time to 200 ms. MS/MS spectra were generated using collision-induced dissociation over a m/zrange from 100 to 2500 of the top three most abundant precursors, applying an isolation width of three Thomson. The fragmentation cut-off was set to 27% with 100% fragmentation amplitude using the Enhanced SmartFrag option from 30 to 120% in 32 ms and ICC was set to 150,000.

### Data Processing and Analysis

The MALDI-FT-ICR MS data were processed using DataAnalysis version 5 (Bruker). Namely, the file, containing the raw data, was split into individual spectra per sample (xy files). For glycan annotation, the mMass (43) software was used and for each of these spectra the calibration was performed using MassyTools (version 2.0.0) (44). The glycan calibrants are shown in supplemental Table S1. The MassyTools output file was further used for analyte curation, which was based on S/N ≥9, isotopic pattern quality ≤0.45, and mass accuracy between ±20 ppm. The glycan compositions passing these criteria in 45% of the total number of samples were taken in consideration for further processing. The peak areas of the curated glycans were calculated for each spectrum using MassyTools and relative abundances were determined by total area normalization. To base the analysis on common structural features, rather than on individual glycans, the glycosylation traits were calculated. The formulae used to compute these from the relative area of the glycan compositions are described in supplemental Table S2. Glycans were abbreviated according to their monosaccharide composition (H = hexose; N = *N*-acetylhexosamine; F = fucose; E or L = *N*-acetylneuraminic acid for α2,6- and α2,3-linked variants respectively; Ge or Gl = *N*-glycolylneuraminic acid for α2,6- and α2,3-linked variants respectively) and the glycan compositions are assigned as [M + Na]^+^ (40).

For PGC nano-LC-ESI-MS/MS the glycan structures were measured as [M – H]^-^ and assigned based on the known MS/MS fragmentation patterns in negative-ion mode (45), elution order, and general glycobiological knowledge, with the help of Glycoworkbench (46) and Glycomod (47) software.

For proteomics, the MS raw data files were converted to mzML format (centroid mode) using the MSconvert tool of the software ProteoWizard (version 3.0.1957). Converted mzML files were then analyzed using OpenMS ver. 2.4 operating within the open-source software platform KNIME ver. 4.1.1 (48). Peptide identification was done using an approach combining the search engines as previously described (49). Briefly, Uniprot FASTA database for the mouse (niport-mus+musculus.fasta, downloaded at www.uniprot.org, Jan 2022, 17.527 entries), and a common contaminant proteins database were used. The spectral library required by the SpectraST search engine was downloaded from the website www.peptideatlas.org (file NIST_mouse_IT_2012-04-21_7AA.splib). Peptide sequences were indexed through the OpenMS PeptideIndexer node, setting leucine/isoleucine equivalence. Except for SpectraST, all search engines set cysteine carbamidomethylation as a fixed modification and methionine oxidation as a variable modification. The tolerance for fragment mass was set at 0.02 Da, and the tolerance for precursor mass was set at 5.0 ppm. The number of missed cleavages permitted in the X!Tandem and MS-GF+ adapters was set to 1. Protein inference was then carried out using the Protein Inference Analysis (PIA) algorithm (50). Protein abundance estimates were calculated with prior generation of spectral feature by the node FeatureFinderMultiplex followed by PIA-assisted FDR-multiple scores estimation and filtering (combined FDR score<0.01), their ID mapping and combination with peptide IDs, their subsequent alignment, grouping and normalization (MapAlignerIdentification, FeatureUnlabeledQT, and ConsensusmapNormalizer nodes) (51). Protein and peptide label-free quantification (LFQ) was then computed with the OpenMS ProteinQuantifier node based on intensities of the n = 3 most abundant identified peptides. The corresponding output files were read as tables of the CSVreader node output and exported into Microsoft Office Excel 2016 for further formatting and statistical elaboration.

### Statistical Analysis

For graphic presentation and statistical analysis, Graph Pad-Prism 8 (GraphPad Software) was used. Results are expressed as the mean per group ± standard deviation (SD). For comparison between the two groups, an unpaired parametric *t* test with a 95% confidence interval was used. For multiple comparisons, the Bonferroni-Dunn method with a *p* < 0.05 was applied (37, 38).

## Results

**Fig. 1:**
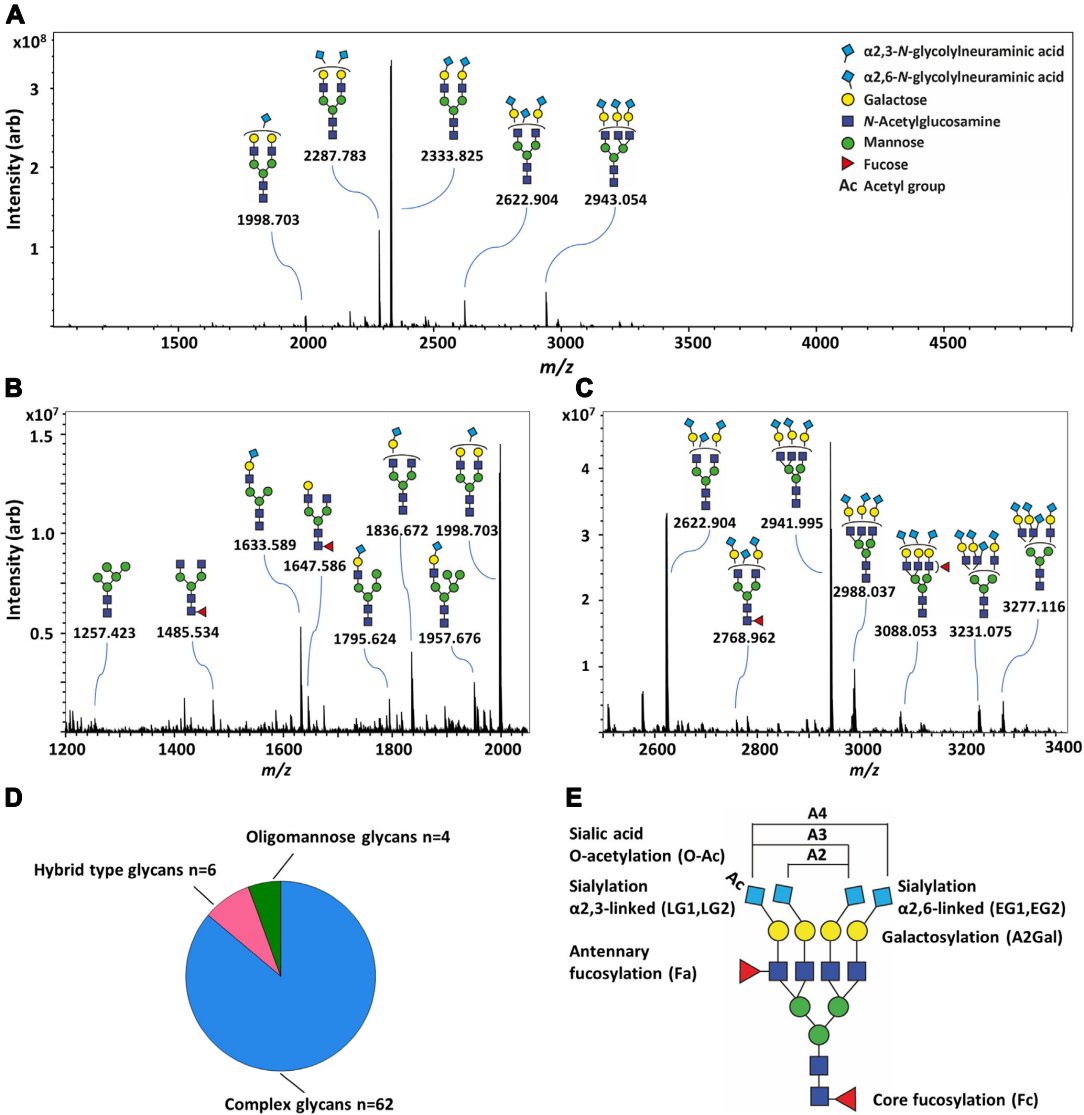
**Exemplary mouse total plasma N-glycome.***A*, MALDI-FT-ICR spectrum of total plasma N-glycome in MRC1-deficient mice. *B*, low mass range from *m/z* 1200 to *m/z* 2060. *C*, high mass range from *m/z* 2500 to *m/z* 3400. Glycans are detected as [M + Na]^+^. Structures are assigned based on PGC-LC-MS (2) and linkage-specific sialic acid derivatization. *D*, compositional assignment of complex, hybrid, and oligomannose glycans in TPNG of MRC1 and ASGR1 deficient mice. *E*, schematic of calculated glycosylation traits.

In this study, we present the TPNG and plasma proteome of ASGR1 and MRC1-deficient mice. ASGR1^−/−^ and MRC1^−/−^ mice showed no signs of residual receptors (supplemental Fig. S1). The N-glycome analysis was performed by MALDI-FTICR-MS, including the differentiation of α2,3- and α2,6-linked Neu5Gc and Neu5Ac by linkage-specific derivatization (41). This derivatization induces a mass difference between the otherwise isomeric structures, allowing differentiation in MS without the need for fragmentation (40). This method allowed a deep profiling of TPNG with high precision as demonstrated on a pool of mouse plasma (Fig. 1, *A*–*C* and supplemental Fig. S2). Profiles of WT mice were highly comparable to previous reports (8). The TPNG profiles were obtained from seven-months-old, male mice – ASGR1^−/−^ (n = 12) *versus* WT (n = 9), and MRC1^−/−^ (n = 10) *versus* WT (n = 8). The mice were sacrificed after 20 weeks of high-fat diet and the data were obtained from three independent experimental groups.

In plasma from both ASGR1^−/−^ and MRC1^−/−^ mice, 72 N-glycan compositions were assigned of which 62 accounted for complex glycans, followed by six hybrid glycans and four oligomannose glycans as shown in Figure 1*D* (8). Compositions are signals with the same elemental composition, mostly containing the same monosaccharides, yet potentially encompassing multiple structural isomers (supplemental Table S3). Additionally, porous graphitized carbon (PGC) LC-MS/MS was performed to obtain (partial) structural assignment of the N-glycans (supplemental Figs. S4, S6 and S7).

15 glycosylation traits were calculated according to common structural glycan features as shown in Figure 1*E* and supplemental Table S2: α2,3-linked sialylation of mono-/diantennary glycans (LG1) and of tri-/tetrantennary glycans (LG2); α2,6-linked sialylation of mono-/diantennary glycans (EG1) and of tri-/tetrantennary glycans (EG2); galactosylation of mono-/diantennary glycans(A2Gal); core fucosylation (Fc); antennary fucosylation (Fa); and sialic acid O-acetylation (O-Ac). Quantitatively, the most prevalent structures were complex glycans (>99%) with almost complete galactosylation, consisting for ca. 80% of diantennary and ca. 20% of triantennary glycans. Hybrid-type glycans and oligomannose glycans accounted for 0.53% and 0.17%, respectively.

### ASGPR Deficiency Increases O-acetylation of Sialic Acid but Does not Increase Terminal Galactosylation

**Fig. 2:**
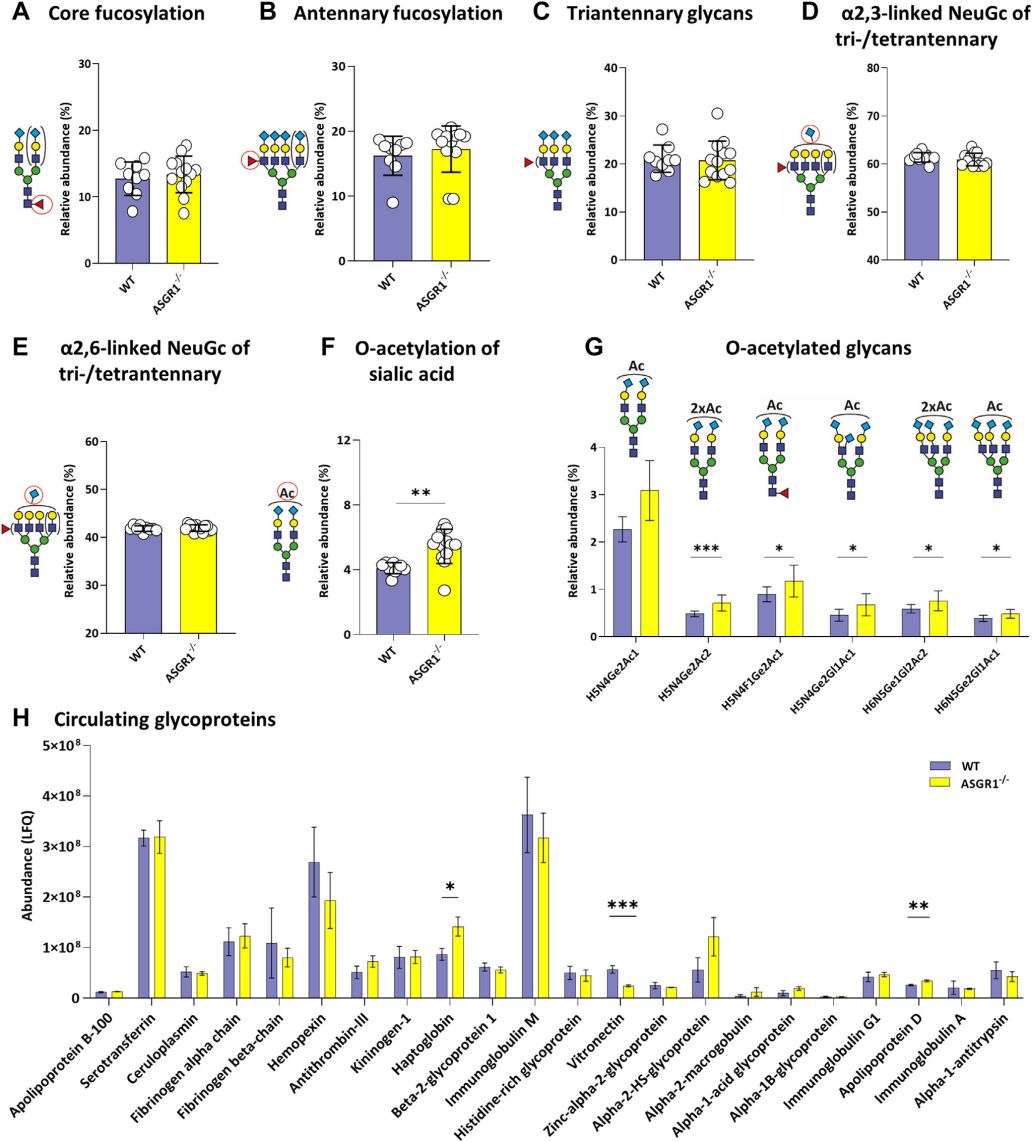
**ASGR1 deficiency results in increased O-acetylation of sialic acid.** Overview of selected glycosylation traits, comparing WT (n = 9) and ASGR1^−/−^ mice (n = 12); mean ± SD depicted for: *A*, Core fucosylation; (*B*) Antennary fucosylation, (*C*) Triantennary glycans, (*D*) α2,3-linked *N*-Glycolylneuraminic acid on tri-/tetraantennary glycans, (*E*) α2,6-linked *N*-Glycolylneuraminic acid on tri-/tetraantennary glycans, (*F*) O-acetylation of sialic acid, and (*G*) O-acetylated glycans. *H*, LFQ of plasma glycoproteins of WT (n = 4) and ASGR1^−/−^ (n = 4) mice. The error bars show the mean ± SD. The proteins are sorted by decreasing confidence based on the number of identified peptides. Non-parametric *t* test was used to compare each group. ∗*p* < 0.05, ∗∗<0.01 and ∗∗∗<0.001.

Given the key role of ASGPR in the clearance of desialylated (and α2,6-sialylated) glycoproteins, we evaluated the terminal galactosylation and α2,3-linked sialylation of complex glycans. It is known that the ligand binding capacity of ASGPR increases from 100- to 1000-fold from mono-to triantennary Gal moieties—unless they are capped with α2,3-linked sialic acid (52). Thus, we expected an accumulation of N-glycans with terminal or α2,6-sialylated galactose and a relative reduction in α2,3-linked sialylation on triantennary glycans, and to a lesser extent on mono-/di-antennary glycans. Surprisingly, neither α2,6-linked sialylation nor α2,3-linked sialylation differed between KO and WT mice, both on mono-/diantennary (supplemental Fig. S3, *H* and *I*) nor on tri-/tetrantennary glycans (Fig. 2, *D* and *E*). Overall, TPNG was remarkably stable upon ASGPR knockout (Fig. 2, *A*–*E*, supplemental Figs. S3 and S4).

Considering that modification with α2,3-linked sialic acid can protect glycoproteins from degradation and clearance, we further evaluated the presence of O-acetylation (O-Ac), as a sialic acid substituent (Fig. 2*F*). ASGR1^−/−^ presented a 33% increase in O-Ac compared to the WT mice (WT 4.1% ± 0.3%, ASGR1^−/−^ 5.45% ± 1.1%, *p*-value<0.002). O-Ac remains an independent finding also after the application of the Bonferroni-Dunn correction (corrected α = 0.0033). Sialic acid can be O-acetylated in different positions—at C-4/7/8/9. We observe an apparent increase in mono-O-acetylated glycans, such as H5N4Ge2Ac1 (36% increase, *p* = 0.002), H5N4F1Ge2Ac1 (31%, *p* = 0.03), H5N4Ge2Gl1Ac1 (48%, *p* = 0.02), H6N5Ge2Gl1Ac1 (24%, *p* = 0.02), as well as in di-O-acetylated glycans, such as H5N4Ge2Ac2 (47%, *p* < 0.001) and H6N5Ge1Gl2Ac2 (28%, *p* = 0.04), in ASGR1-deficient mice compared to WT (Fig. 2*G*). All O-acetylated compositions also contain at least one α2,6-linked sialic acid which is in line with earlier reports of ST6Gal1 involvement and observation of diagnostic ions for acetylated α2,6-linked NeuGc (7, 8). We observed structures with up to three O-Ac per α2,6-linked NeuGc.

Shotgun plasma proteomics of ASGR1^−/−^ mice showed a significant increase in haptoglobin (HPT) and apolipoprotein D (ApoD) compared with their WT littermates (Fig. 2*H*, supplemental Table S4). A similar increase in HPT abundance has been reported previously (53). Therein, it has also been discussed that saturation of the receptor and competition between ligands play a crucial role. HPT, a glycoprotein with four N-glycosylation sites occupied by complex N-glycans, is involved in binding free plasma hemoglobin to allow hepatic recycling of heme iron (54). In mice, HPT has been reported to be decorated with highly sialylated di- and tri-antennary glycans (55). However, treatment with α2,3-sialidase of haptoglobin showed that, at least in humans, the sialic acids were mainly α2,6-linked, suggesting that HPT is a substrate for ASGPR (35, 53). Apolipoprotein D, a glycoprotein with two glycosylation sites, with sialylated triantennary or fucosylated sialylated biantennary complex glycans is associated with high-density lipoprotein (HDL), by forming a complex with the enzyme bound to HDL known as Phosphatidylcholine-Sterol O-Acyltransferase (56, 57). Although the biological function of ApoD and its glycosylation remains unknown, whether changes in N-glycosylation might modulate ApoD protein folding and binding to specific ligands within HDL, should be further investigated (58). Moreover, vitronectin, a glycoprotein with three N-glycosylation sites, is significantly downregulated in ASGR1^−/−^ compared to WT mice. Vitronectin is known to be decreased in serum during liver conditions such as fatty liver, steatohepatitis, fibrosis, and cirrhosis (59). It has been reported to contain highly sialylated di- and triantennary glycans in healthy volunteers (60). Unfortunately, the glycosylation of apolipoprotein D and vitronectin in mice is not reported with sufficient molecular resolution to add to the discussion. Furthermore, O-acetylation data for the individual proteins was not available. Therefore, the connections between glycomics and proteomics data, we have drawn, have to be considered extrapolations. Nonetheless, similarities are significant between human and mouse plasma N-glycome as well as between the glycosylation of the few investigated major plasma proteins, for example, IgG, in these species (39, 55, 61).

### MRC1 Deficiency Affects Fucosylation but not Oligomannose Abundance

**Fig. 3:**
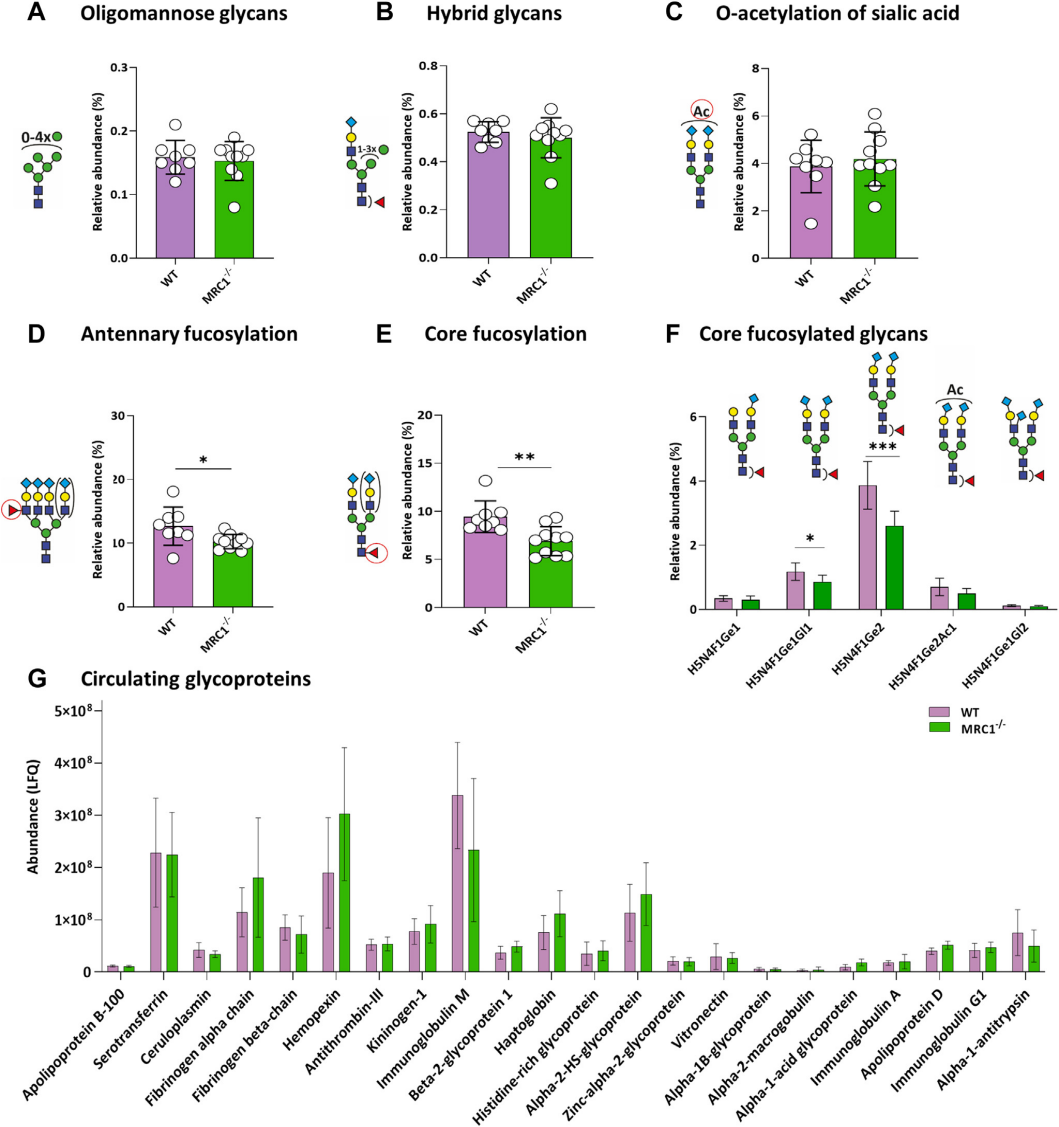
**MRC1 deficiency results in decreased core fucosylation.** Overview of selected glycosylation traits, comparing WT (n = 8) and MRC1^−/−^ mice (n = 10); mean ± SD depicted for: (*A*) Oligomannose glycans, (*B*) Hybrid glycans, (*C*) O-acetylation of sialic acid, (*D*) Antennary fucosylation, (*E*) Core fucosylation, and (*F*) Core fucosylated glycans. *G*, LFQ of plasma glycoproteins in WT (n = 4) and MRC1^−/−^ (n = 4) mice. The error bars show the mean ± SD. The proteins are sorted by decreasing confidence based on the number of identified peptides. Non-parametric *t* test was used to compare each group. ∗*p* < 0.05, ∗∗<0.01 and ∗∗∗<0.001.

Contrary to expectation, the oligomannose preference of MRC1 did not lead to an increase in oligomannose glycans in the TPNG profiles of MRC1 deficient mice in comparison with their WT controls (Fig. 3*A*). Like the ASGPR knockout, also the MRC1 knockout had a surprisingly limited effect on TPNG composition (Fig. 3, *A*–*C*, supplemental Figs. S5 and S6). However, MRC1-deficient mice did present a 27% reduction in core fucosylation (WT 9.5% ± 1.6%, MRC1^−/−^ 6.9% ± 1.5%, *p*-value=0.003; Fig. 3*E*) and a 19% reduction in antennary fucosylation (WT 12.7% ± 3.0%, MRC1^−/−^ 10.2% ± 1.1%, *p* = 0.03; Fig. 3*D*). The main glycans contributing to Fc were H5N4F1Ge1 (13% reduction, n.s.), H5N4F1Ge1Gl1 (27%, *p* = 0.01, H5N4F1Ge2 (33%, *p* = 0.0004), H5N4F1Ge2Ac1 (28%, n.s.), and H5N4F1Ge1Gl2 (23%, n.s.) (Fig. 3*F*). The main glycans contributing to Fa were H6N5F1Ge1Gl2 (32% reduction, *p* = 0.01), H6N5F1Ge1Gl2Ac1 (36%, *p* = 0.01), H6N5F1Ge2Gl1 (33%, *p* = 0.006) (supplemental Fig. S6). The reduction in both Fc and Fa combined clearly shows that there is an effect on fucosylation. However, the t-tests were not conclusive as to whether core or antennary fucosylation or both are specifically affected—no independent findings after multiple testing corrections (corrected α = 0.0033). Of note, core fucosylation was calculated as the fucosylation of mono- and diantennary glycans. In contrast, antennary fucosylation was calculated as the fucosylation of tri- and tetraantennary glycans. This is a good approximation for antennary fucosylation in TPNG, supported by literature (62).

## Discussion

C-type lectins, prominently the asialoglycoprotein receptor (ASGPR) and the mannose receptor C-type 1 (MRC1), contribute to the clearance of glycoproteins in a glycoform-specific manner. Though this is well established for individual proteins, such as therapeutic antibodies, the contribution to and impact on serum/plasma protein homeostasis is less well understood. Especially, there have been (partially) contradicting studies for ASGPR while MRC1 impact has not been assessed for the major serum/plasma glycoproteins at all. However, understanding the impact of these lectin clearance pathways is important to the design and quality of therapeutic glycoproteins, to understanding the effects of disturbances in glycosylation in disease, as well as to fully comprehending glycoprotein turnover. To address this, we mapped changes in the major plasma glycoproteins on the level of the total plasma N-glycome (TPNG) as well as the serum proteome upon the knockout of these two prominent clearance receptors. Since glycome and proteome are highly sensitive to mouse strain differences and environmental variability, we took great care to maximize group comparability for the animal experiments. For example, plasma from ASGR1^−/−^ or MRC1^−/−^ mice was compared with their respective littermate WT controls, which were housed in the same room. Surprisingly, we found a very limited impact of the knockouts on both TPNG as well as the serum proteome.

### ASGPR Deficiency may be Compensated by Redundant Receptors

The affinity of ASGPR and its role in glycoprotein clearance would suggest more terminal or α2,3-sialylated galactose on glycoproteins in deficient mice (5). However, we observed no relative changes in these features and a similar lack of accumulation of glycoproteins with terminal Gal has been reported in other studies (22). This has sometimes been attributed to the presence of α2,6-sialylated structures. However, given our linkage-specific detection, we can rule out this explanation. An alternative explanation is the presence of functionally redundant receptors, relevant for glycoprotein clearance and overlapping in specificity with ASGPR. These receptors may be able to take over the task of clearing glycoproteins with a terminal or α2,6-sialylated Gal motif. A well-described candidate for such ASGPR redundancy is a C-type lectin receptor known as CLEC4F or Kupffer cell receptor which has a specificity overlapping with that of ASGPR (63, 64).

In contrast to the general absence of change, we observed a relative accumulation of O-acetylation (O-Ac) of sialic acid. Such changes have not been reported previously. This is most likely explained by the inability of most alternative methods to assess this highly labile modification. O-Ac can be modulated by the availability of the sialylated glycan acceptor and by the availability of the acetyl group donor (65). Interestingly, the acetyl donor is acetyl-coenzyme A (Acetyl-CoA), the key molecule in cellular metabolism for energy production (7). O-Ac hinders the enzymatic activity of circulating sialidases. For example, Hunter and coworkers showed that 9-O-acetylation (Neu5,9Ac_2_) reduced the activity of NEU3 (66, 67). Thus, increased O-Ac in ASGR1^−/−^ potentially decreases the hydrolysis of sialic acid on glycoproteins. Since this would decrease the availability of terminal galactose on glycoproteins and thus substrate availability for the putative alternative clearance receptors, this is in line with earlier observations of the increased half-life of circulating glycoproteins upon ASGPR knock-out (22). We also observed a relative increase in the abundance of HTP, a likely ASGPR target, in the plasma of ASGPR deficient mice which would be consistent with a selectively longer half-life. Unfortunately, it is not known whether HTP carriers O-Ac.

### Limited Impact of MRC1 Deficiency may be Explained by the Inaccessibility of Oligomannose Glycans or Redundant Receptors

Lee *et al.* (24) reported that glycoproteins with accessible mannose and GlcNAc residues are cleared slower within the liver of MRC1^−/−^ mice compared to the WT. We observed neither a change in the relative amount of oligomannose glycans (Fig. 3*A*), in protein abundance (supplemental Table S5) nor in the average number of mannoses (data not shown). Therefore, we suspect an inaccessibility of oligomannose-carrying glycosylation sites on major plasma glycoproteins. IgM, a major contributor to oligomannose glycans in human TPNG, also carries about 5% oligomannose structures in mice (35, 68). Indeed, the two sites mainly carrying oligomannose glycans, N279 (N402) and N439/N440 (N563), are buried deep in the IgM pentamer (69).

Furthermore, accessible oligomannose sites should be rapidly trimmed down to Man5 by circulating mannosidase 1—a 1A and 1B variant exist—while the oligomannose portion of TPNG is dominated by Man9 (70, 71). Further processing to complex-type glycans, which was equally unaffected, is performed by mannosidase 2 (72). If a glycosylation site becomes inaccessible during protein folding at an early stage of processing it is likely to carry oligomannose glycans. On the other hand, many pathogenic microorganisms carry oligomannose or high mannose glycans which are targeted by pattern-recognition receptors, such as MRC1 (9). Consequently, there could be evolutionary pressure to reduce accessible oligomannose-bearing glycosylation sites on plasma glycoproteins to avoid self-recognition.

The lower fucosylation observed in the MRC1-deficient mice is equally counterintuitive. While the cysteine-rich domain has been reported to bind fucose in the context of Lewis-type structures—featuring α1,2-, α1,3-, and/or α1,4-linked fucosylation (10, 12)—an important contribution of MRC1 to the clearance of Lewis structures should result in a higher fucosylation of MRC1-deficient mice. Furthermore, our data also indicates a decrease in core fucosylation which is exclusively α1,6-linked and thus cannot be affected by the Lewis structure affinity. In addition, no change was observed in fucosylated ligands by Lee *et al.* (24), suggesting that MRC1 is not essential for the clearance of all fucosylated ligands. In the same work, an increase in eight lysosomal hydrolases including α-mannosidase, β-galactosidase, and α-fucosidase was observed. The decreased fucosylation might therefore be due to the increased circulation of α-fucosidase. Regarding increased circulating α-mannosidase and β-galactosidase in MRC1 deficiency models, it does not surprise that no corresponding changes in TPNG glycosylation are observed: For one, it is unclear whether their increased lysosomal levels translate into increased levels in the circulation. Moreover, the conditions in the circulation including pH may not favor their activity. Also, TPNG may be considered a poor target for these enzymes: Poor accessibility of TPNG oligomannosidic glycans was already discussed above. Terminal galactoses in TPNG, necessary for β-galactosidase activity, are mainly contributed by the Fc glycosylation site of immunoglobulin G which is known for its limited accessibility due to its position in the grove between the two heavy chains (35, 73, 74). In fact, the removal of galactose from IgG Fc N-glycans has never been reported, despite various relevant studies on therapeutic monoclonal antibodies (75).

Altogether, our results rule out that a conserved oligomannose pattern appears in MRC1 deficiency independently of the phenotype differences previously reported (76). Considering the importance of mannose as a pattern recognition target in the immune system and in the clearance of glycoproteins, it is also likely that redundant receptors may be able to take over part of its function in the absence of MRC1. CTL candidates with mannose-binding affinity are described, namely, mannose-binding lectin (MBL) (77), Macrophage C-type lectin (MCL) (78, 79), Macrophage inducible C-type lectin (MINCLE) (79) and Dendritic Cell-Specific Intercellular adhesion molecule-3-Grabbing Non-integrin (DC-SIGN) (80).

## Conclusion

Our study suggests that tight control of plasma protein N-glycosylation and clearance is so important for an organism that significant redundancy exists in terms of plasma glycoprotein clearance receptors with glycan-epitope specificity. Despite the loss of what are considered the two major glycoprotein clearance receptors, TPNG, and the plasma proteome, hardly changed in signature.

Several CTLs could lead to the biological redundancy of this system which is suggested by its stability. For example, CLEC4F for ASGPR and DC-SIGN were mentioned in the Discussion (63, 64, 80). It remains to be explored to what extent they are involved in the clearance of plasma glycoproteins or whether there are additional mechanisms stabilizing the composition of TPNG and the plasma proteome. Studying knockout models of these putatively redundant receptors, using the workflows described herein, should provide evidence of their involvement and relative importance in this specific aspect of protein turnover.

Furthermore, the role of glycan remodeling, triggered by ASGPR deficiency, and its role in the half-life increase of (exogenous) glycoproteins warrants additional investigation. Transcriptional and/or translational information on the enzymes involved in the O-acetylation of sialic acids in the liver and plasma of this model should be very informative. Alternatively, administration and glycosylation monitoring of recombinant proteins that feature O-Ac, such as erythropoietin, would allow us to dissect whether the observed increase in O-Ac is due to disturbances in glycoprotein biosynthesis or due to changes in clearance/glycan remodeling from/in plasma.

## Data Availability

The proteomics data have been deposited to the ProteomeXchange Consortium *via* PRIDE repository with reference number PXD042269. Glycomics MALDI-FTICR and PGC-MS/MS data were added to GlycoPost under the identifier GPST000351. Annotated glycan MS/MS spectra are available as supplementary material.

## Supplemental data

This article contains supplemental data.

## Conflict of interest

The authors declare that they have no known competing financial interests or personal relationships that could have appeared to influence the work reported in this paper.
