# Supplementary material for: Impact of Asialoglycoprotein Receptor and Mannose Receptor Deficiency on Murine Plasma N-glycome Profiles
Source: Mol Cell Proteomics. 2023 Jul 4;22(9):100615. doi: 10.1016/j.mcpro.2023.100615 (PMC10462831; doi:10.1016/j.mcpro.2023.100615)
Supplement: Supplemental Figure S7 [file mmc1.pptx]

## Slide 1
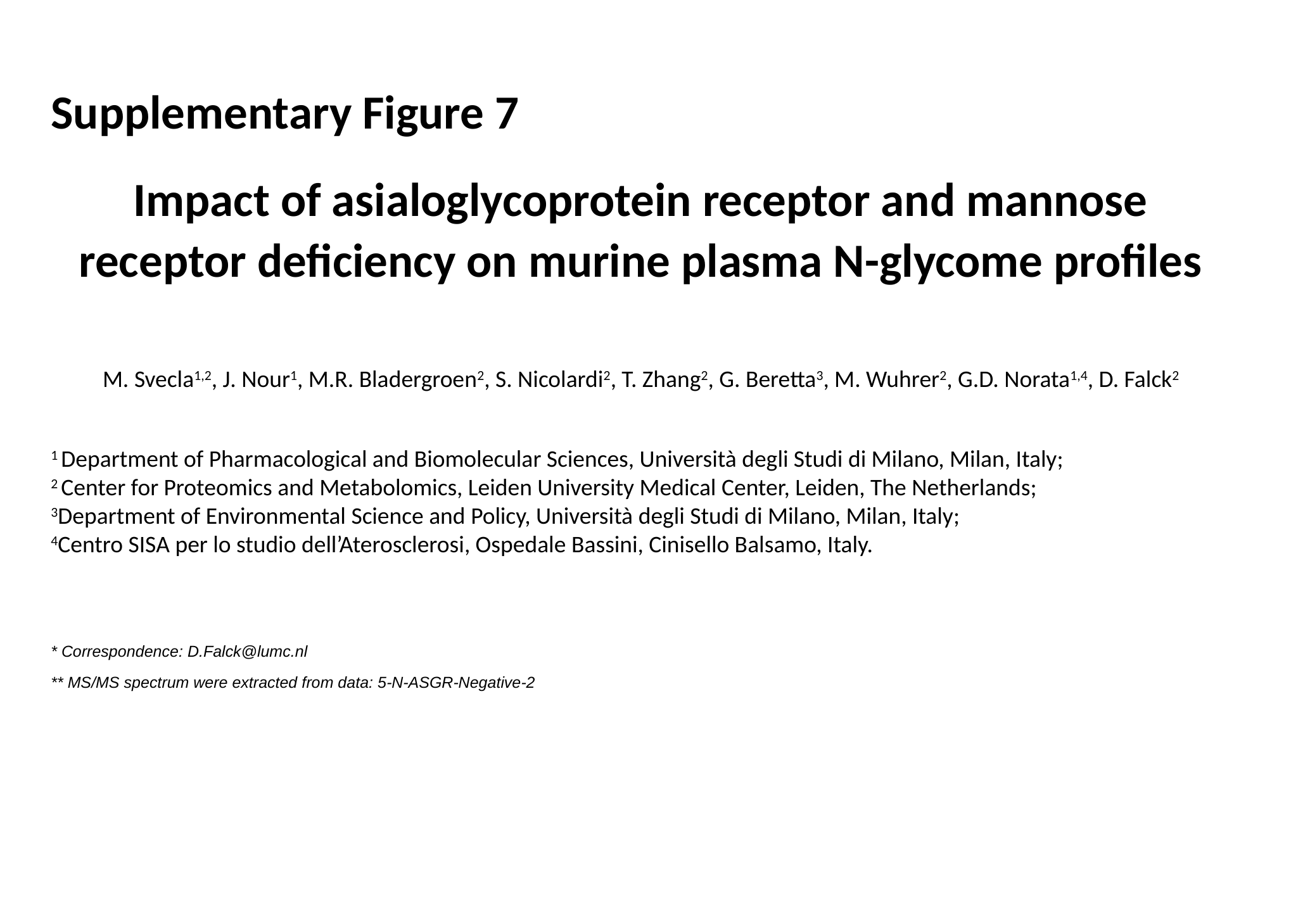

Supplementary Figure 7
Impact of asialoglycoprotein receptor and mannose receptor deficiency on murine plasma N-glycome profiles
M. Svecla1,2, J. Nour1, M.R. Bladergroen2, S. Nicolardi2, T. Zhang2, G. Beretta3, M. Wuhrer2, G.D. Norata1,4, D. Falck2
1 Department of Pharmacological and Biomolecular Sciences, Università degli Studi di Milano, Milan, Italy;
2 Center for Proteomics and Metabolomics, Leiden University Medical Center, Leiden, The Netherlands;
3Department of Environmental Science and Policy, Università degli Studi di Milano, Milan, Italy;
4Centro SISA per lo studio dell’Aterosclerosi, Ospedale Bassini, Cinisello Balsamo, Italy.
* Correspondence: D.Falck@lumc.nl
** MS/MS spectrum were extracted from data: 5-N-ASGR-Negative-2

## Slide 2
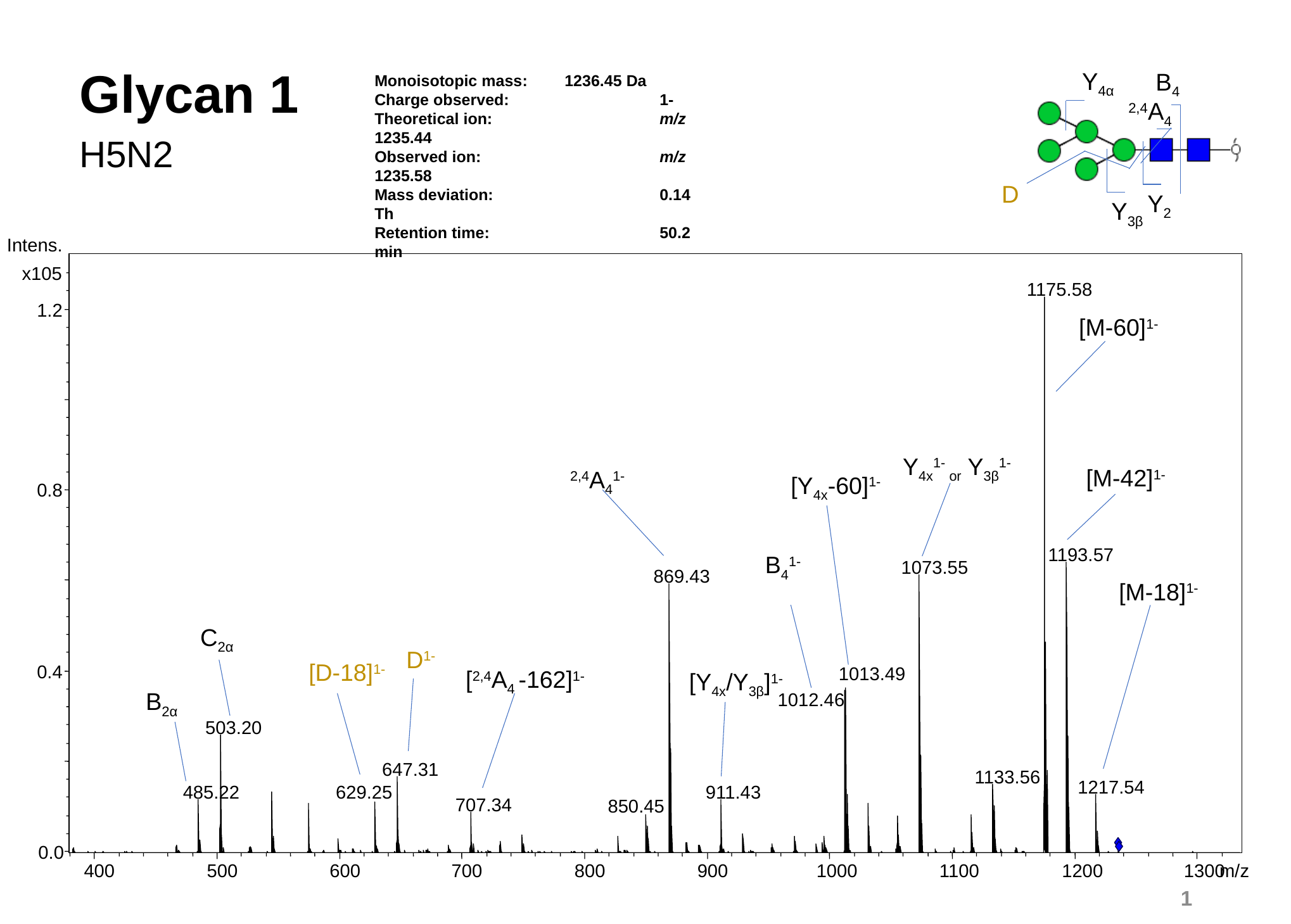

Glycan 1
Y4α
B4
Monoisotopic mass:	1236.45 Da
Charge observed:		1-
Theoretical ion: 		m/z 1235.44
Observed ion: 		m/z 1235.58
Mass deviation:		0.14 Th
Retention time: 		50.2 min
H5N2
2,4A4
Y2
Y3β
D
Intens.
x105
1175.58
1.2
0.8
1193.57
1073.55
869.43
0.4
1013.49
503.20
647.31
1133.56
1217.54
485.22
629.25
911.43
707.34
850.45
0.0
400
500
600
700
800
900
1000
1100
1200
1300
m/z
[M-60]1-
Y4x1- or Y3β1-
[M-42]1-
2,4A41-
[Y4x-60]1-
B41-
[M-18]1-
C2α
D1-
[D-18]1-
[2,4A4 -162]1-
[Y4x/Y3β]1-
B2α
1012.46
2

## Slide 3
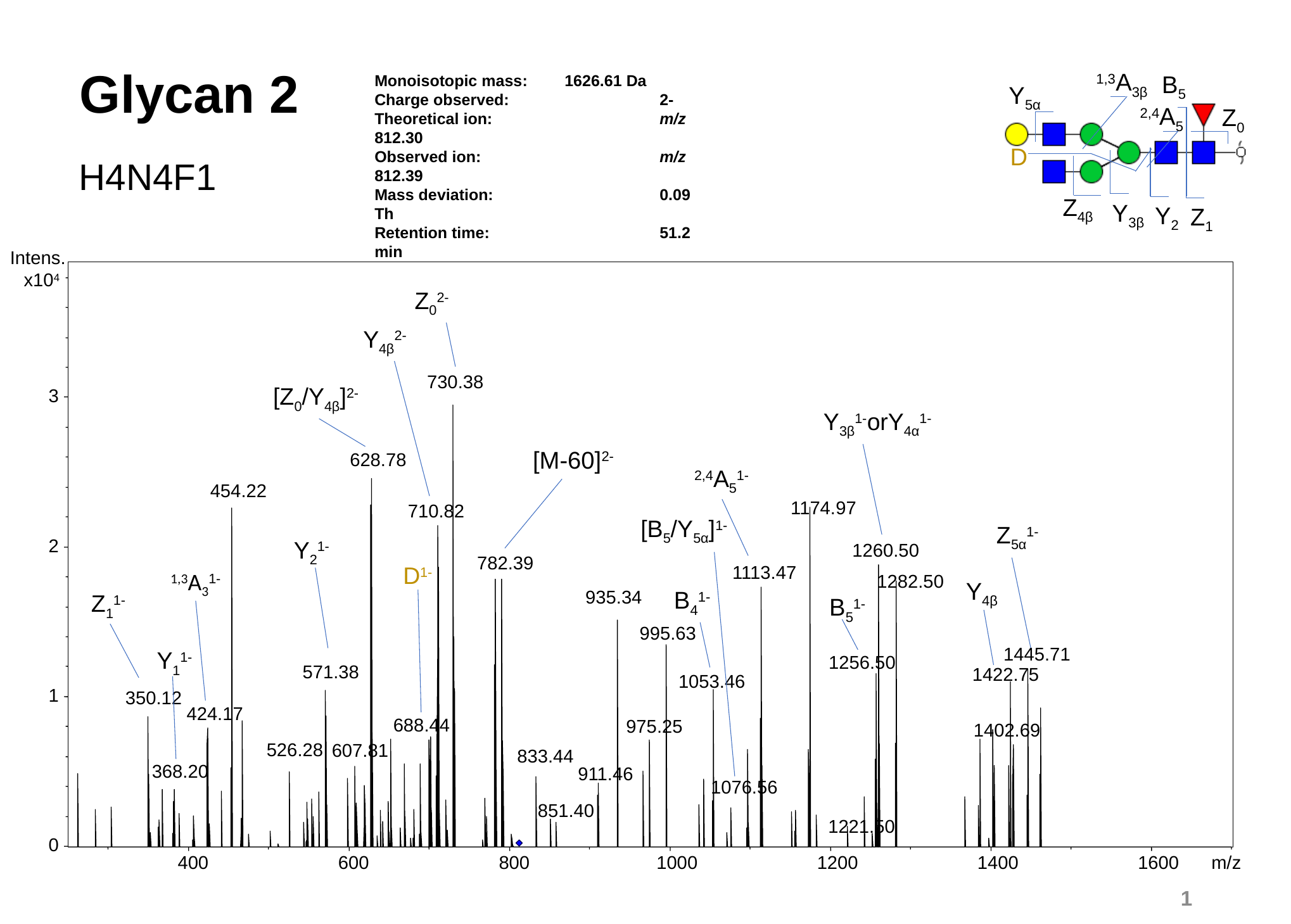

Glycan 2
1,3A3β
B5
Monoisotopic mass:	1626.61 Da
Charge observed:		2-
Theoretical ion: 		m/z 812.30
Observed ion: 		m/z 812.39
Mass deviation:		0.09 Th
Retention time: 		51.2 min
Y5α
H4N4F1
2,4A5
Z0
D
Y2
Z1
Y3β
Z4β
Intens.
x104
730.38
3
628.78
454.22
1174.97
710.82
2
1260.50
782.39
1113.47
1282.50
935.34
995.63
1445.71
571.38
1422.75
1053.46
1
350.12
424.17
688.44
975.25
1402.69
526.28
607.81
833.44
911.46
1076.56
851.40
1221.50
0
400
600
800
1000
1200
1400
1600
m/z
Z02-
Y4β2-
[Z0/Y4β]2-
Y3β1-orY4α1-
[M-60]2-
2,4A51-
[B5/Y5α]1-
Z5α1-
Y21-
D1-
1,3A31-
Y4β
B41-
Z11-
B51-
Y11-
1256.50
368.20
3

## Slide 4
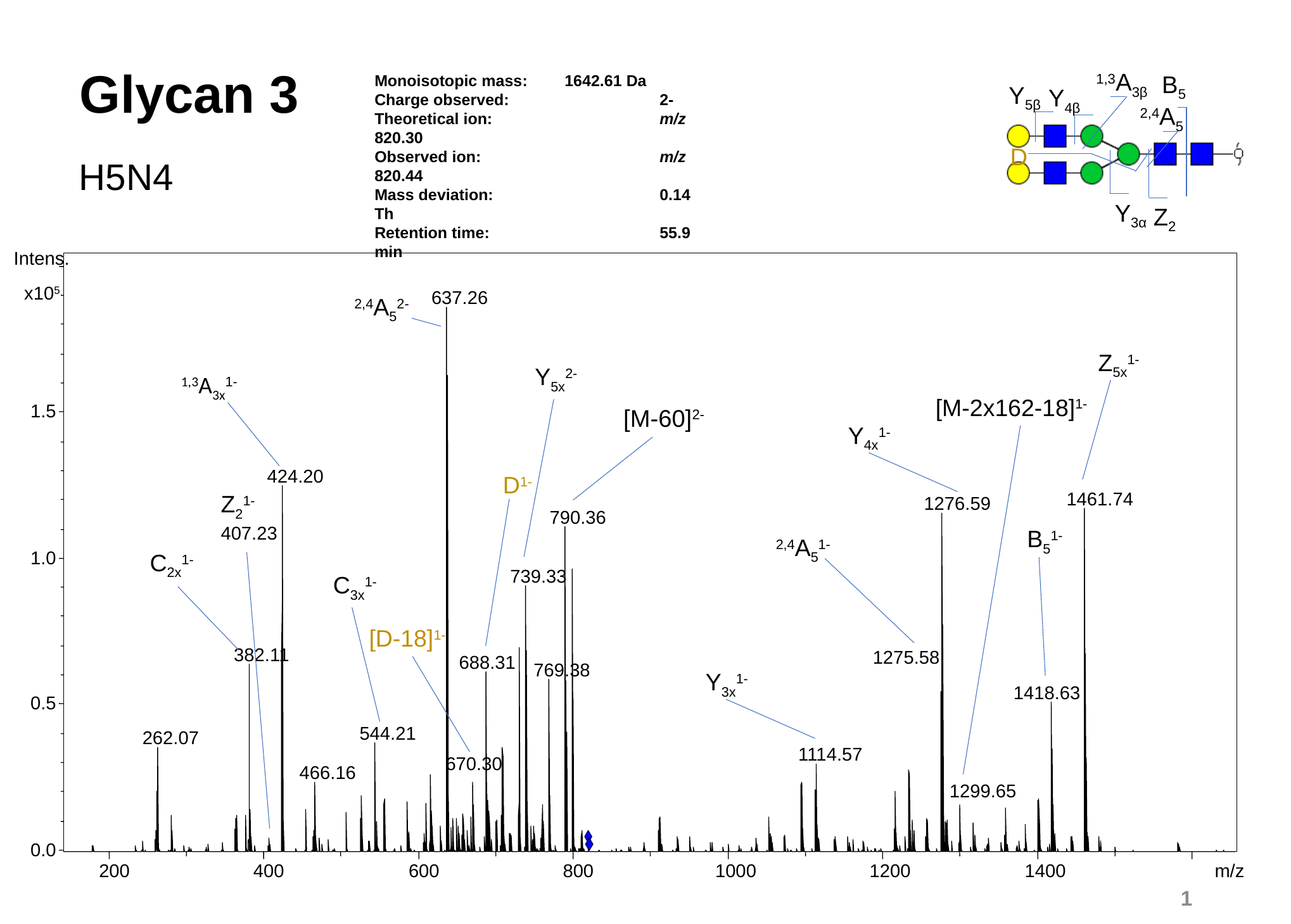

Glycan 3
1,3A3β
B5
Monoisotopic mass:	1642.61 Da
Charge observed:		2-
Theoretical ion: 		m/z 820.30
Observed ion: 		m/z 820.44
Mass deviation:		0.14 Th
Retention time: 		55.9 min
Y5β
Y4β
H5N4
2,4A5
D
Z2
Y3α
Intens.
x105
637.26
1.5
424.20
1461.74
1276.59
790.36
1.0
739.33
382.11
1275.58
688.31
769.38
1418.63
0.5
544.21
262.07
1114.57
466.16
1299.65
0.0
200
400
600
800
1000
1200
1400
m/z
2,4A52-
Z5x1-
Y5x2-
1,3A3x1-
[M-2x162-18]1-
[M-60]2-
Y4x1-
D1-
Z21-
407.23
B51-
2,4A51-
C2x1-
C3x1-
[D-18]1-
Y3x1-
670.30
4

## Slide 5
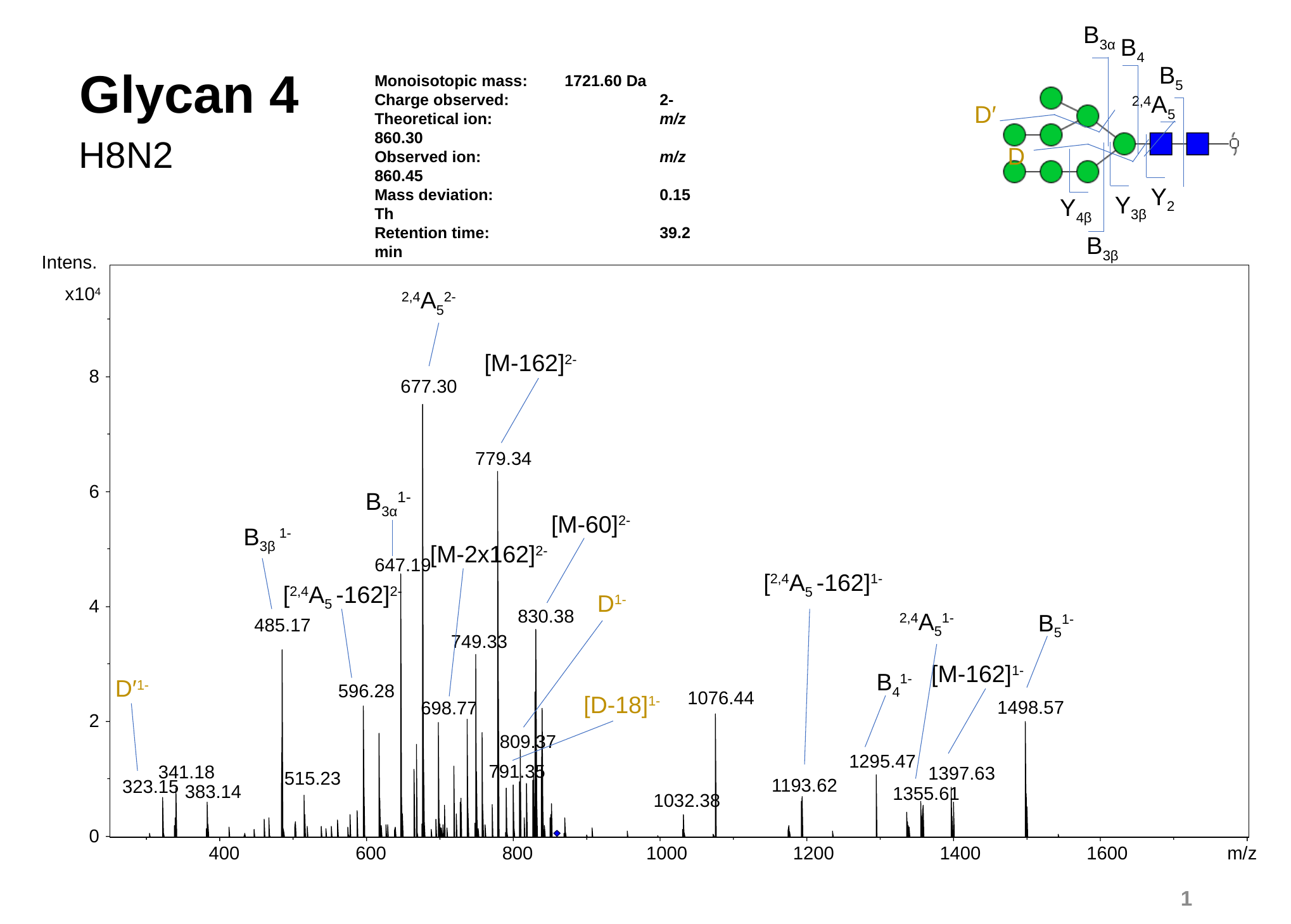

B3α
B4
Glycan 4
B5
Monoisotopic mass:	1721.60 Da
Charge observed:		2-
Theoretical ion: 		m/z 860.30
Observed ion: 		m/z 860.45
Mass deviation:		0.15 Th
Retention time: 		39.2 min
2,4A5
H8N2
D′
Y2
D
Y3β
Y4β
B3β
Intens.
x104
8
677.30
779.34
6
647.19
4
830.38
485.17
749.33
596.28
1076.44
1498.57
698.77
2
809.37
1295.47
341.18
1397.63
515.23
1193.62
383.14
1355.61
1032.38
0
400
600
800
1000
1200
1400
1600
m/z
2,4A52-
[M-162]2-
B3α1-
[M-60]2-
B3β 1-
[M-2x162]2-
[2,4A5 -162]1-
[2,4A5 -162]2-
D1-
2,4A51-
B51-
[M-162]1-
B41-
D′1-
[D-18]1-
791.35
323.15
5

## Slide 6
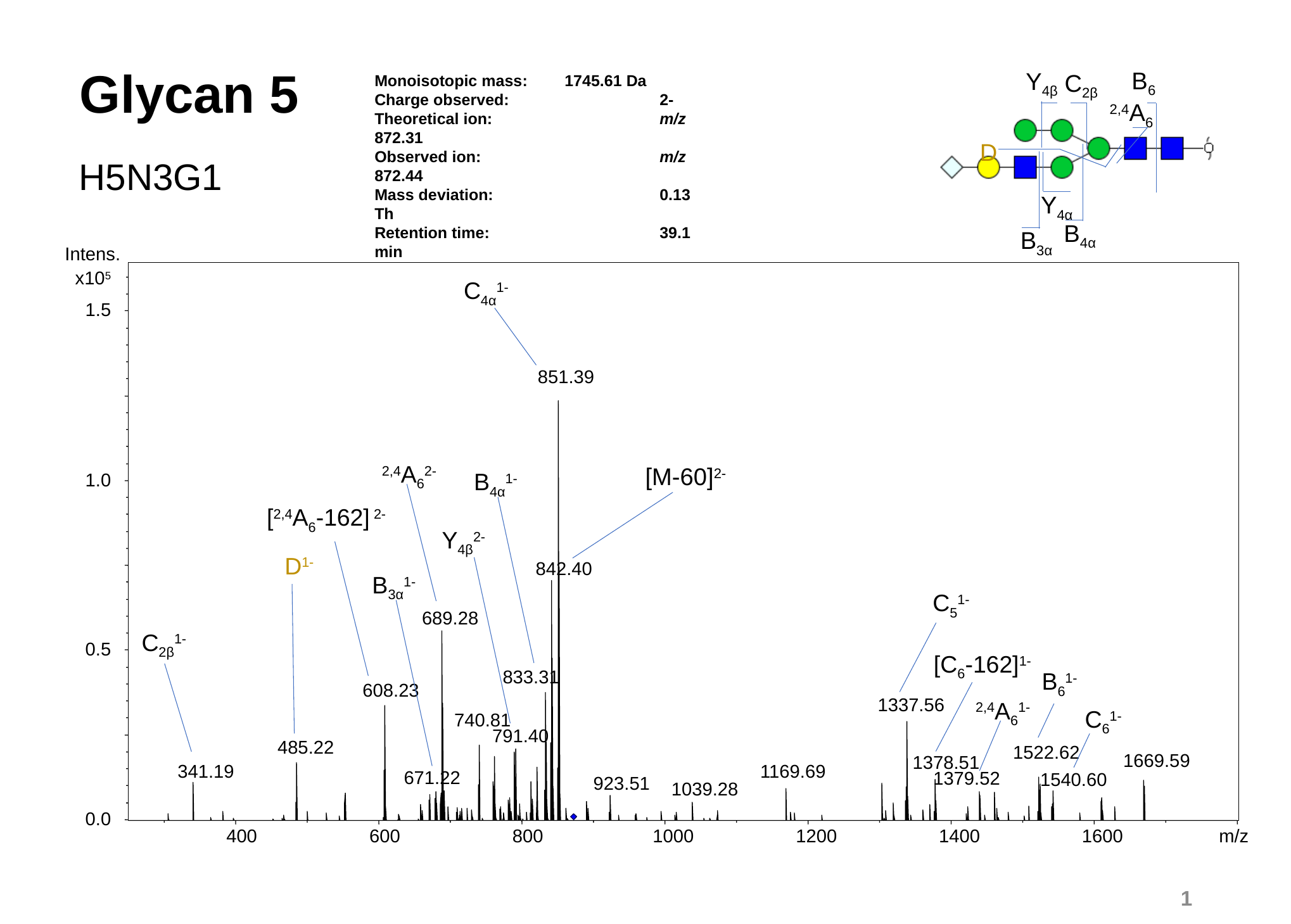

Glycan 5
B6
Y4β
C2β
Monoisotopic mass:	1745.61 Da
Charge observed:		2-
Theoretical ion: 		m/z 872.31
Observed ion: 		m/z 872.44
Mass deviation:		0.13 Th
Retention time: 		39.1 min
H5N3G1
2,4A6
D
B4α
B3α
Y4α
Intens.
x105
1.5
851.39
1.0
689.28
0.5
608.23
1337.56
740.81
791.40
485.22
1522.62
1669.59
1378.51
341.19
1169.69
671.22
1540.60
923.51
1039.28
0.0
400
600
800
1000
1200
1400
1600
m/z
C4α1-
2,4A62-
[M-60]2-
B4α1-
[2,4A6-162] 2-
Y4β2-
D1-
842.40
B3α1-
C51-
C2β1-
[C6-162]1-
B61-
833.31
2,4A61-
C61-
1379.52
6

## Slide 7
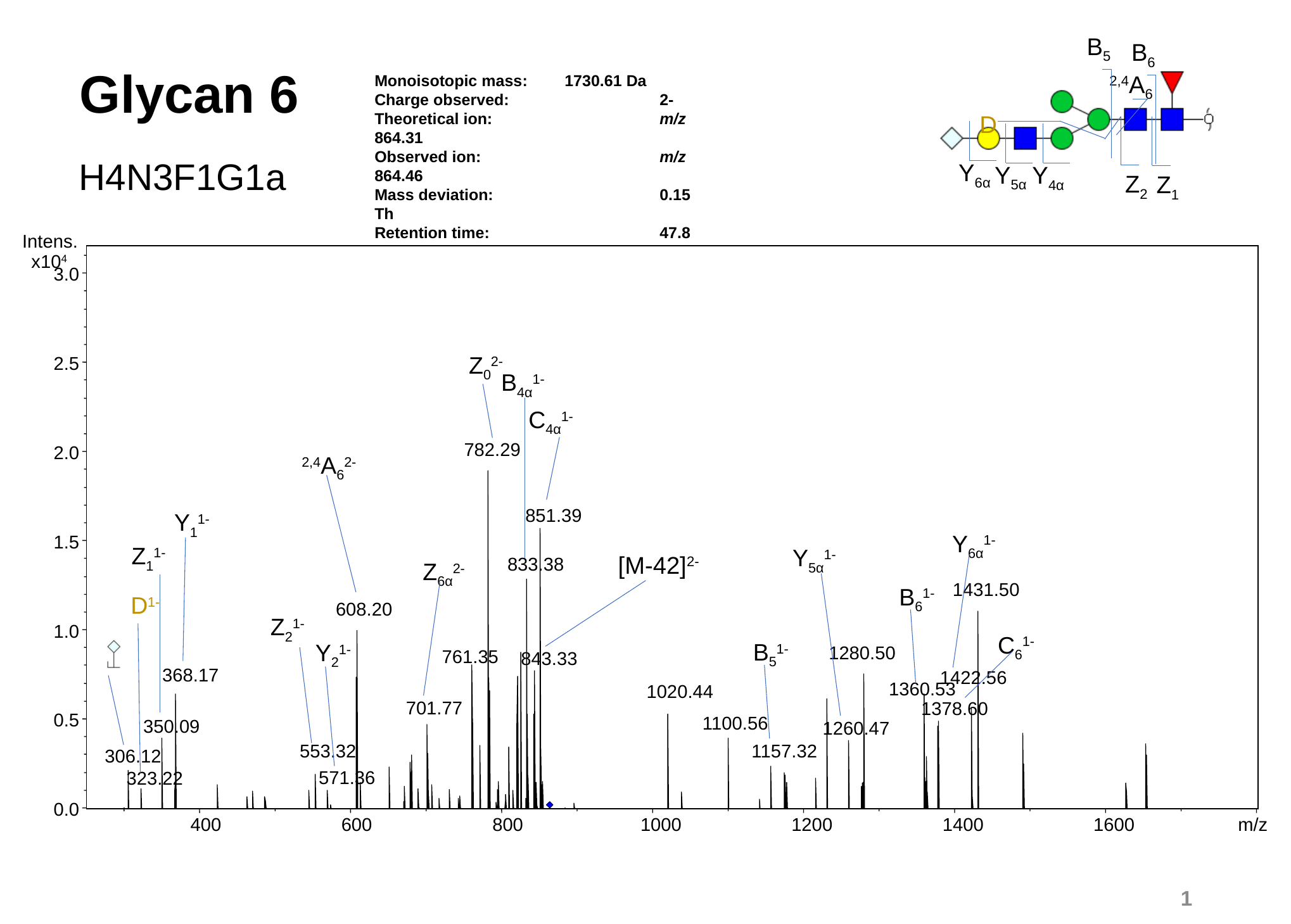

B5
Glycan 6
B6
2,4A6
Monoisotopic mass:	1730.61 Da
Charge observed:		2-
Theoretical ion: 		m/z 864.31
Observed ion: 		m/z 864.46
Mass deviation:		0.15 Th
Retention time: 		47.8 min
H4N3F1G1a
D
Z2
Z1
Y6α
Y5α
Y4α
Intens.
x104
3.0
2.5
782.29
2.0
851.39
1.5
1431.50
608.20
1.0
1280.50
761.35
368.17
1422.56
1360.53
1020.44
701.77
1378.60
0.5
1100.56
1260.47
553.32
1157.32
306.12
571.36
323.22
0.0
400
600
800
1000
1200
1400
1600
m/z
Z02-
B4α1-
C4α1-
2,4A62-
Y11-
Y6α1-
Z11-
Y5α1-
[M-42]2-
Z6α2-
833.38
B61-
D1-
Z21-
C61-
B51-
Y21-
843.33
350.09
7

## Slide 8
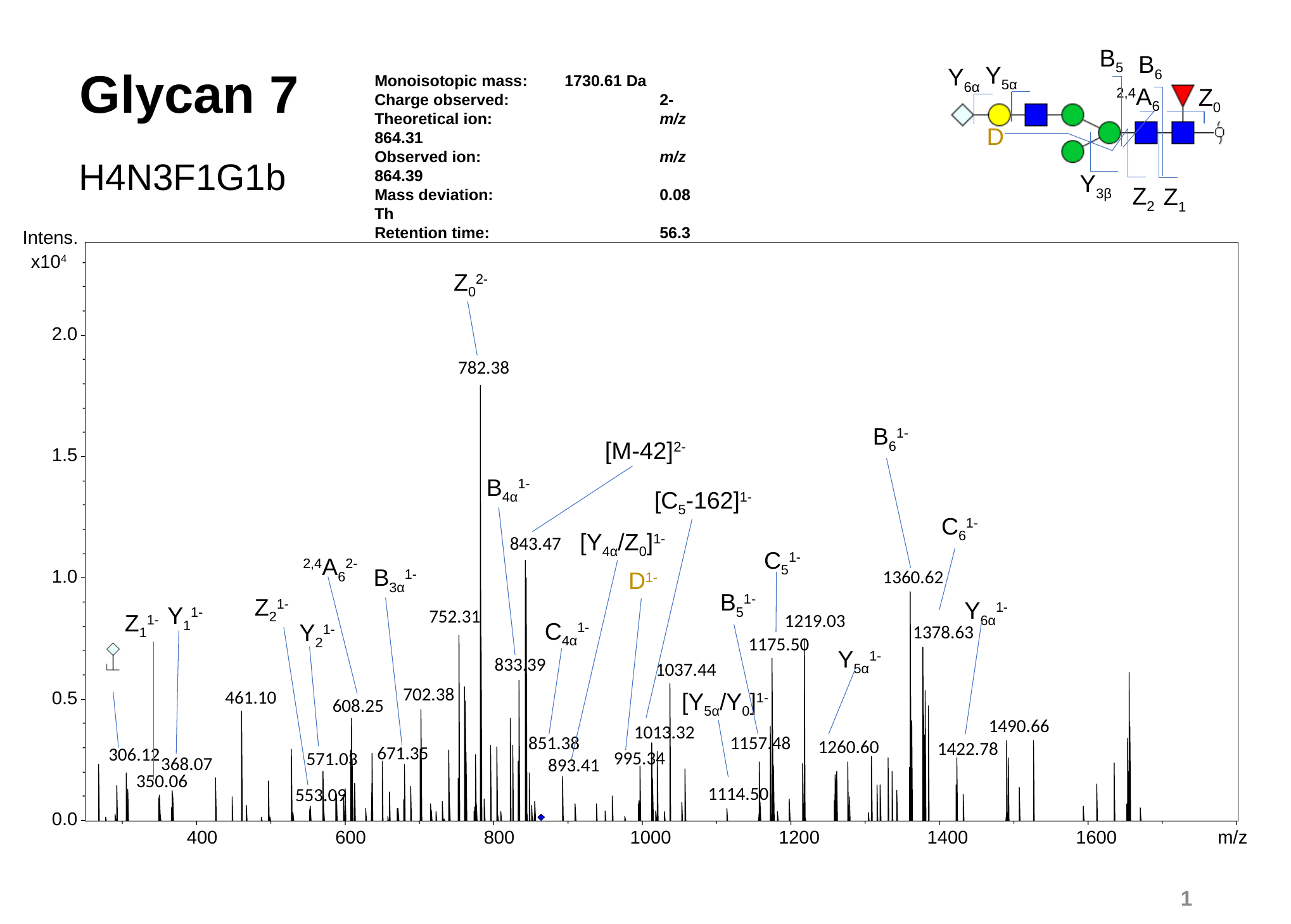

Glycan 7
B5
B6
Y5α
Y6α
Monoisotopic mass:	1730.61 Da
Charge observed:		2-
Theoretical ion: 		m/z 864.31
Observed ion: 		m/z 864.39
Mass deviation:		0.08 Th
Retention time: 		56.3 min
2,4A6
Z0
H4N3F1G1b
D
Z2
Z1
Y3β
Intens.
x104
Z02-
2.0
782.38
B61-
[M-42]2-
1.5
B4α1-
[C5-162]1-
C61-
[Y4α/Z0]1-
843.47
C51-
2,4A62-
B3α1-
D1-
1.0
1360.62
B51-
Z21-
Y6α1-
Y11-
Z11-
752.31
1219.03
C4α1-
Y21-
1378.63
1175.50
Y5α1-
833.39
1037.44
[Y5α/Y0]1-
702.38
461.10
0.5
608.25
1490.66
1013.32
1157.48
851.38
1260.60
1422.78
671.35
306.12
995.34
571.03
368.07
893.41
350.06
1114.50
553.09
0.0
400
600
800
1000
1200
1400
1600
m/z
8

## Slide 9
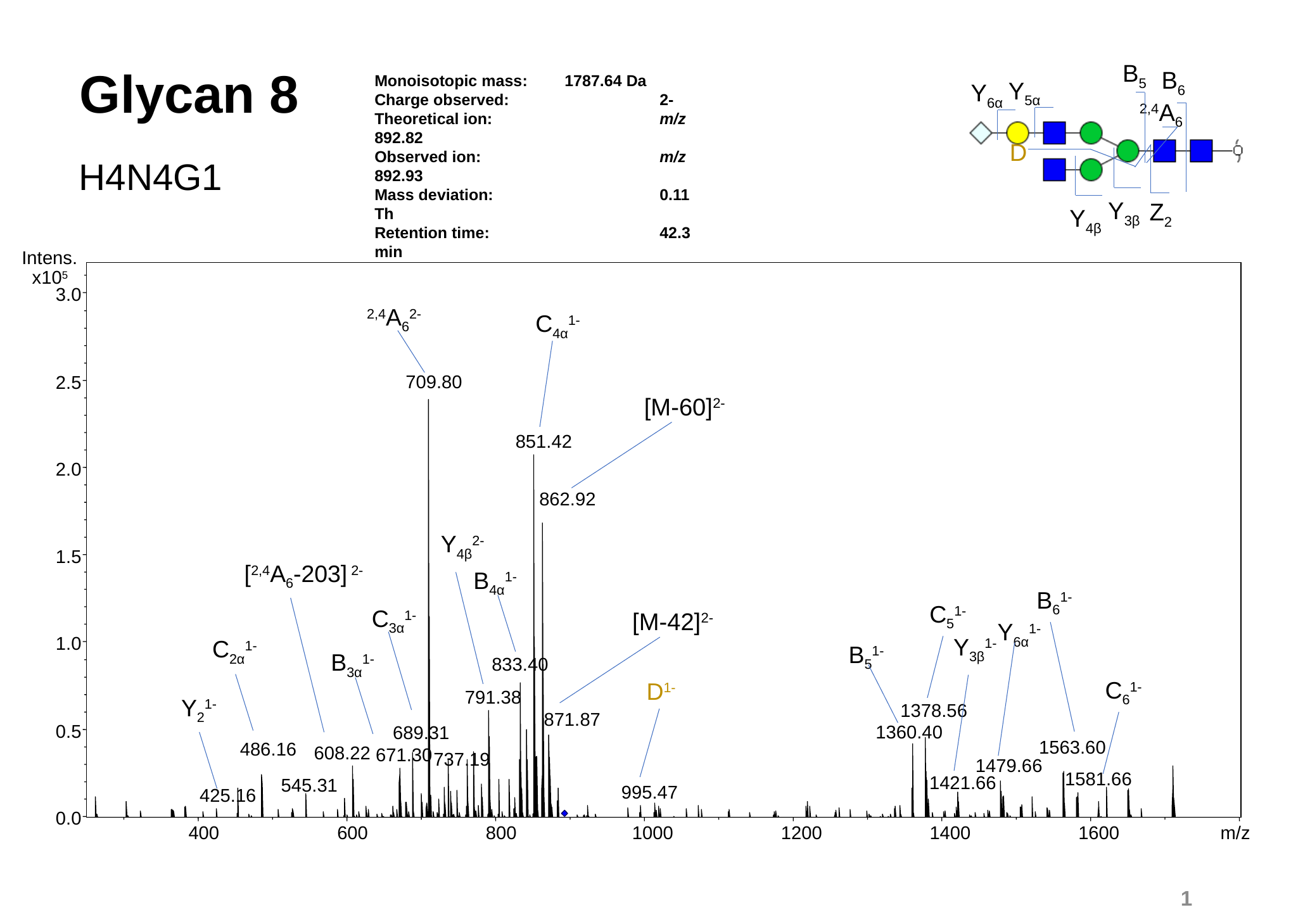

Glycan 8
B5
B6
Monoisotopic mass:	1787.64 Da
Charge observed:		2-
Theoretical ion: 		m/z 892.82
Observed ion: 		m/z 892.93
Mass deviation:		0.11 Th
Retention time: 		42.3 min
Y5α
Y6α
H4N4G1
2,4A6
D
Z2
Y3β
Y4β
Intens.
x105
3.0
709.80
2.5
851.42
2.0
1.5
1.0
791.38
1378.56
0.5
1360.40
689.31
1563.60
486.16
608.22
671.30
737.19
1479.66
1581.66
1421.66
545.31
995.47
425.16
0.0
400
600
800
1000
1200
1400
1600
m/z
2,4A62-
C4α1-
[M-60]2-
862.92
Y4β2-
[2,4A6-203] 2-
B4α1-
B61-
C51-
C3α1-
[M-42]2-
Y6α1-
Y3β1-
C2α1-
B51-
B3α1-
833.40
C61-
D1-
Y21-
871.87
9

## Slide 10
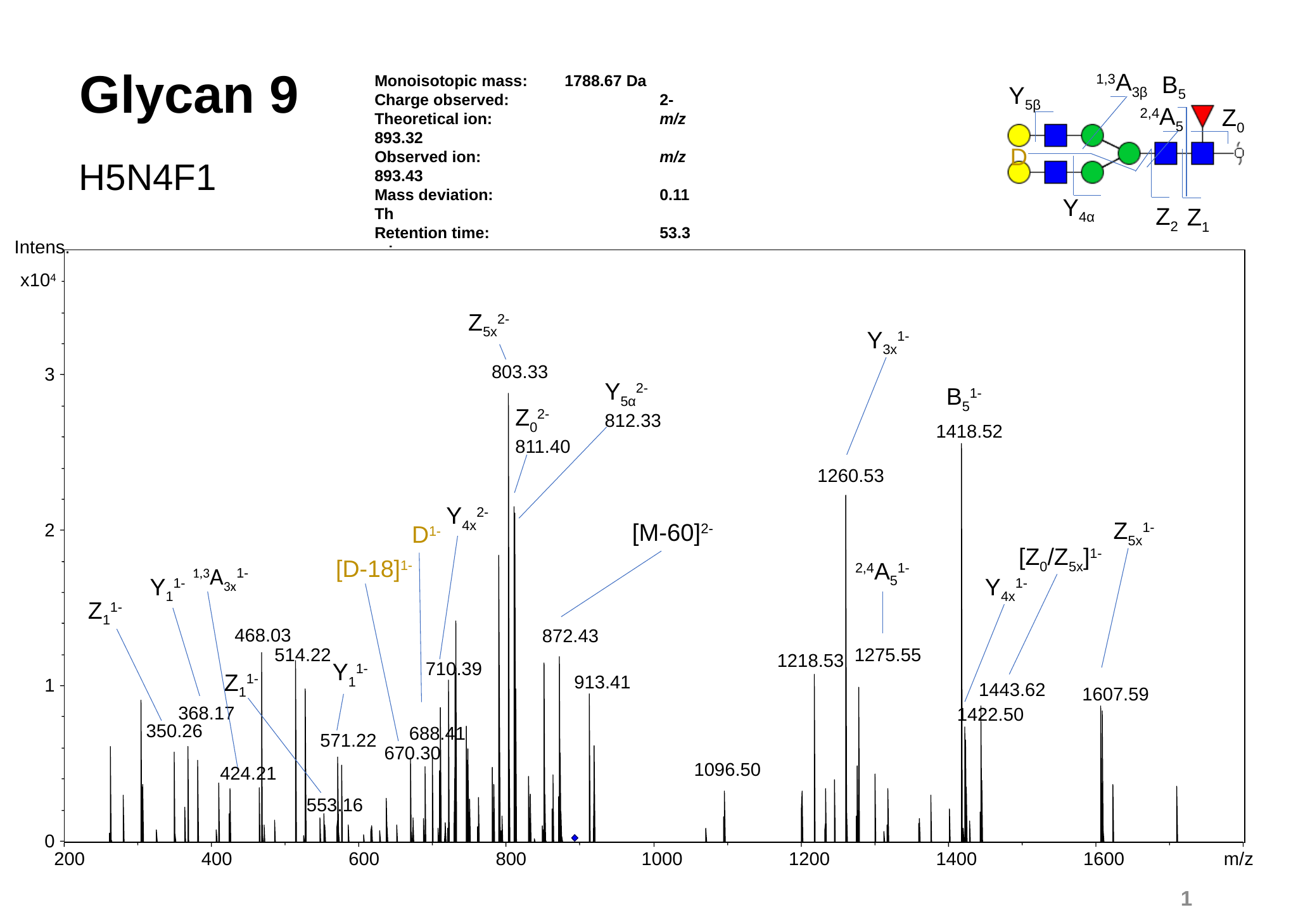

Glycan 9
1,3A3β
B5
Monoisotopic mass:	1788.67 Da
Charge observed:		2-
Theoretical ion: 		m/z 893.32
Observed ion: 		m/z 893.43
Mass deviation:		0.11 Th
Retention time: 		53.3 min
Y5β
H5N4F1
2,4A5
Z0
D
Z2
Z1
Y4α
Intens.
x104
803.33
3
1418.52
1260.53
2
468.03
872.43
514.22
1218.53
710.39
913.41
1
1443.62
1607.59
368.17
350.26
688.41
571.22
670.30
1096.50
424.21
553.16
0
200
400
600
800
1000
1200
1400
1600
m/z
Z5x2-
Y3x1-
Y5α2-
812.33
B51-
Z02-
811.40
Y4x2-
Z5x1-
[M-60]2-
D1-
[Z0/Z5x]1-
[D-18]1-
2,4A51-
1,3A3x1-
Y11-
Y4x1-
Z11-
1275.55
Y11-
Z11-
1422.50
10

## Slide 11
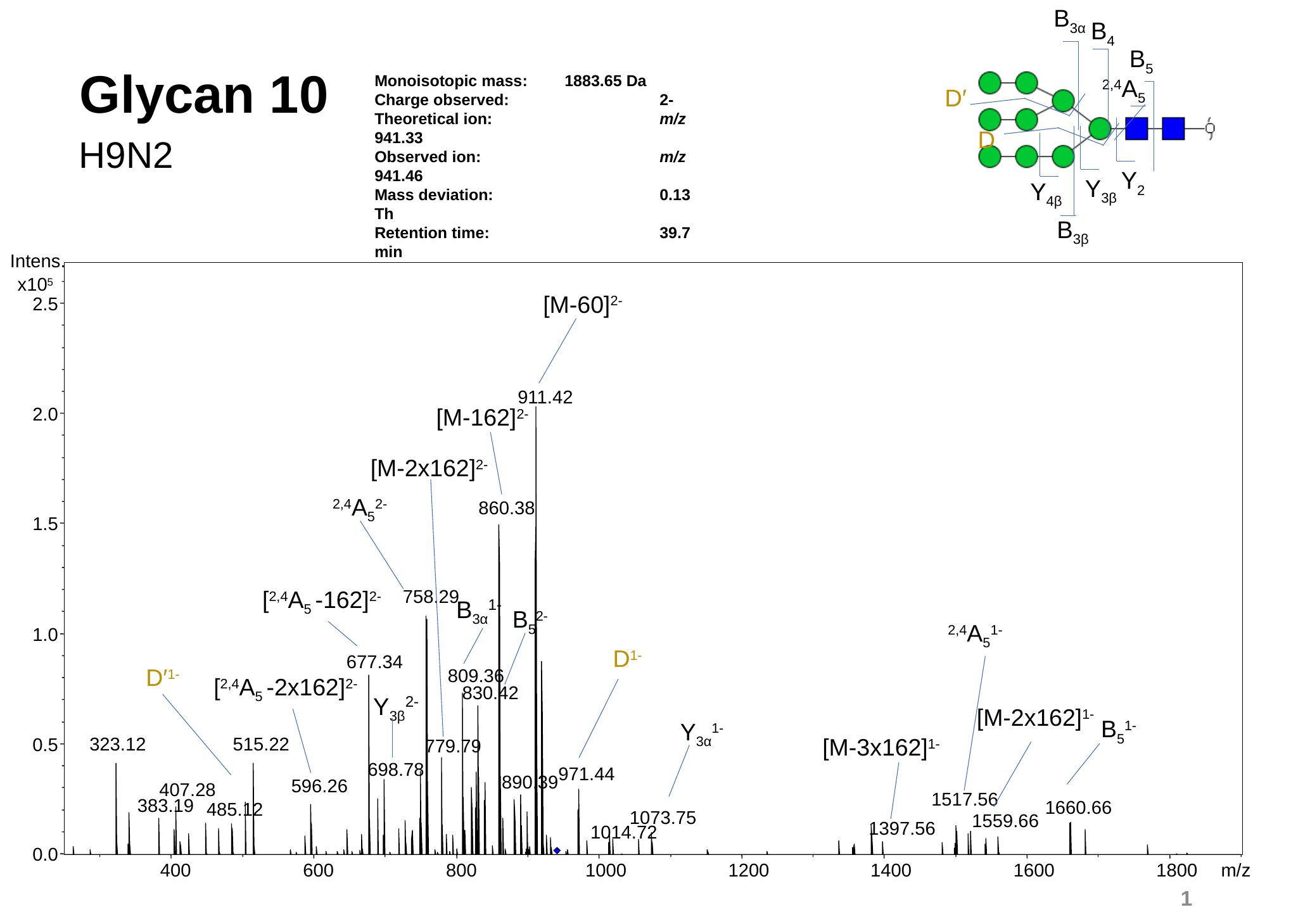

B3α
B4
Glycan 10
B5
Monoisotopic mass:	1883.65 Da
Charge observed:		2-
Theoretical ion: 		m/z 941.33
Observed ion: 		m/z 941.46
Mass deviation:		0.13 Th
Retention time: 		39.7 min
2,4A5
D′
H9N2
Y2
D
Y3β
Y4β
B3β
Intens.
x105
2.5
911.42
2.0
860.38
1.5
758.29
1.0
677.34
809.36
830.42
515.22
323.12
0.5
779.79
698.78
971.44
890.39
596.26
407.28
1517.56
383.19
1660.66
485.12
1073.75
1559.66
1397.56
1014.72
0.0
400
600
800
1000
1200
1400
1600
1800
m/z
[M-60]2-
[M-162]2-
[M-2x162]2-
2,4A52-
[2,4A5 -162]2-
B3α1-
B52-
2,4A51-
D1-
D′1-
[2,4A5 -2x162]2-
Y3β2-
[M-2x162]1-
B51-
Y3α1-
[M-3x162]1-
11

## Slide 12
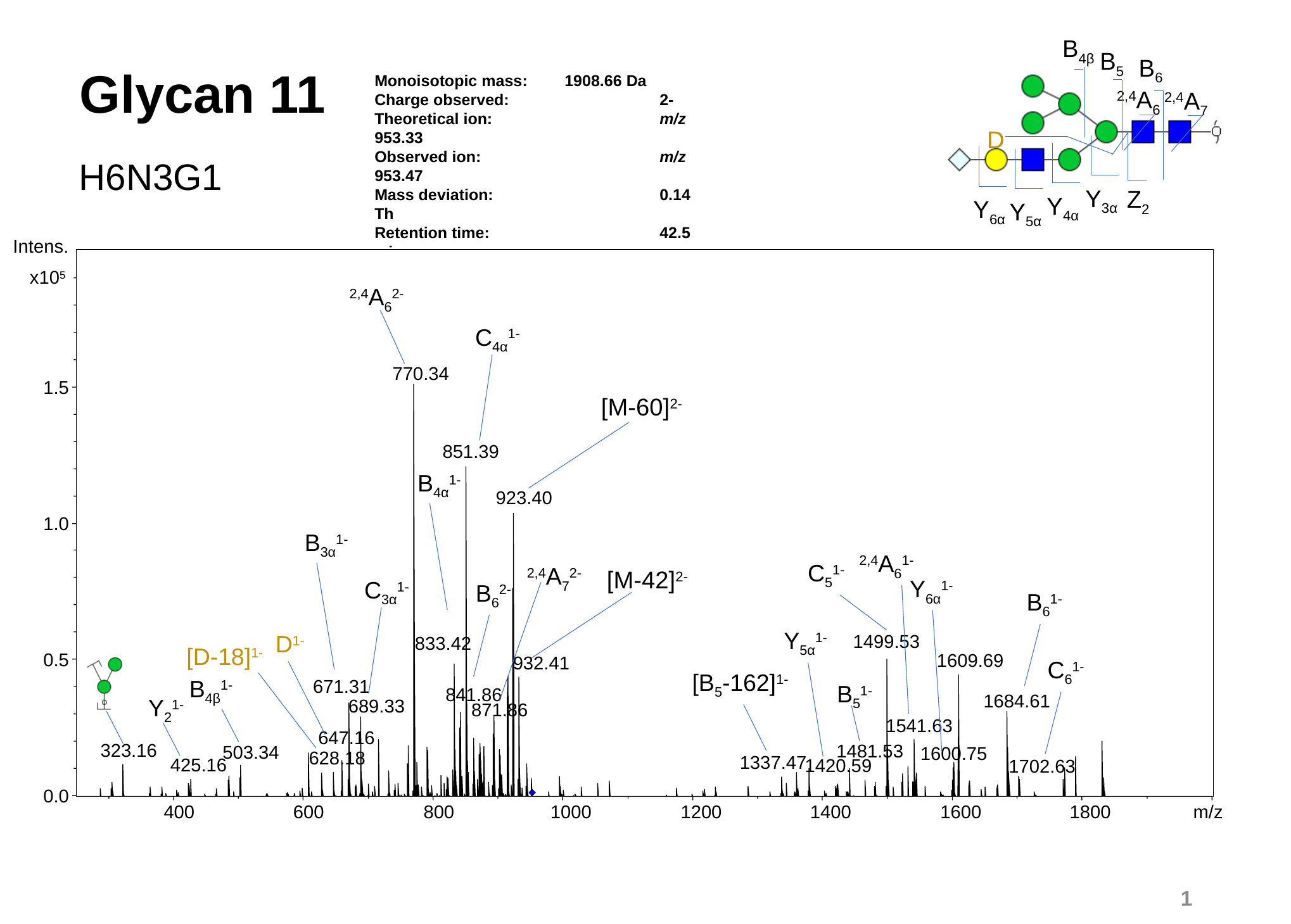

Glycan 11
B4β
B5
B6
Monoisotopic mass:	1908.66 Da
Charge observed:		2-
Theoretical ion: 		m/z 953.33
Observed ion: 		m/z 953.47
Mass deviation:		0.14 Th
Retention time: 		42.5 min
2,4A6
2,4A7
H6N3G1
D
Z2
Y3α
Y4α
Y6α
Y5α
Intens.
x105
770.34
1.5
851.39
923.40
1.0
1499.53
833.42
0.5
1609.69
932.41
671.31
841.86
1684.61
689.33
1541.63
647.16
323.16
1481.53
503.34
1600.75
1337.47
425.16
1420.59
1702.63
0.0
400
600
800
1000
1200
1400
1600
1800
m/z
2,4A62-
C4α1-
[M-60]2-
B4α1-
B3α1-
2,4A61-
C51-
2,4A72-
[M-42]2-
Y6α1-
C3α1-
B62-
B61-
Y5α1-
D1-
[D-18]1-
C61-
[B5-162]1-
B4β1-
B51-
Y21-
871.86
628.18
12

## Slide 13
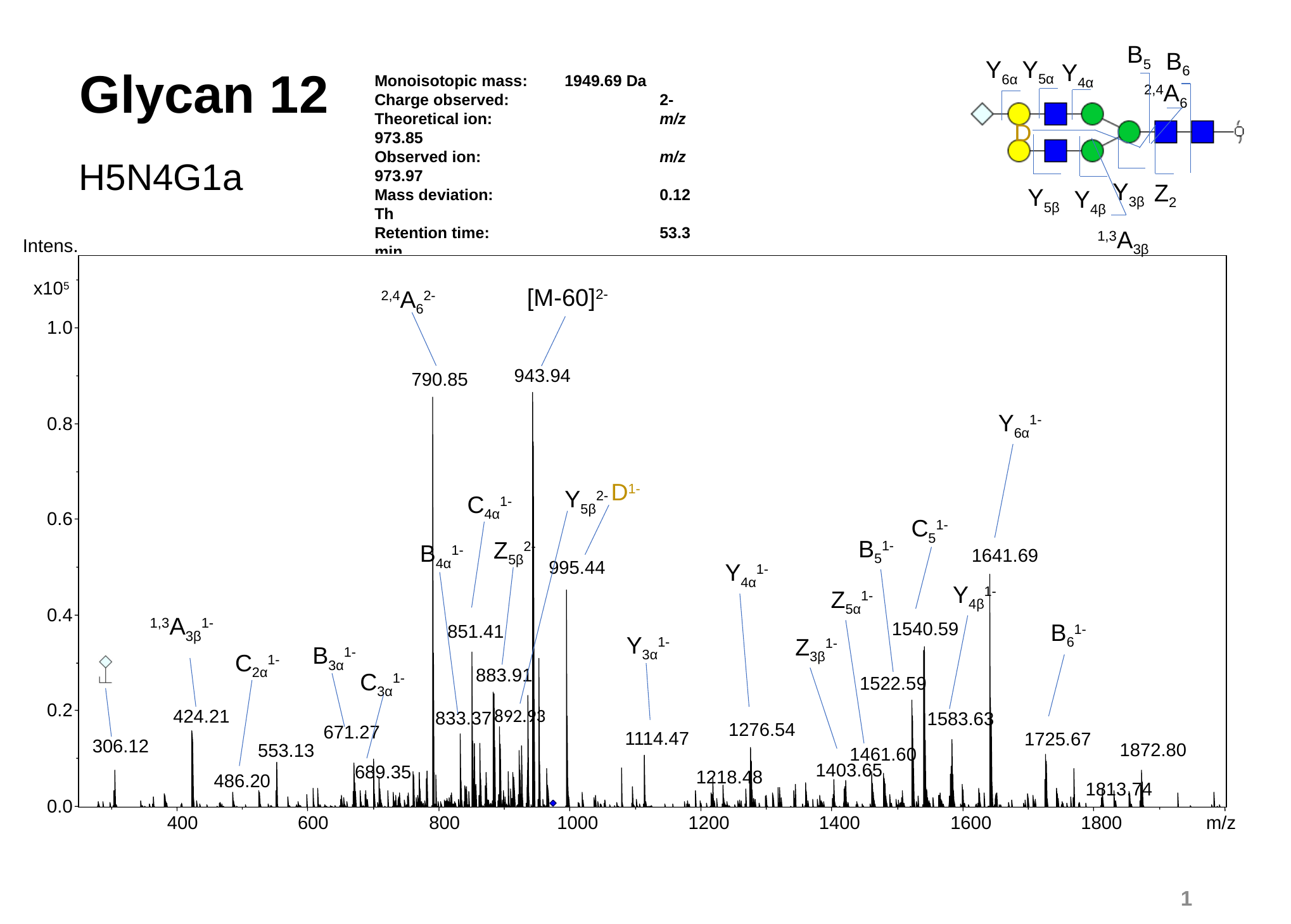

Glycan 12
B5
B6
Y5α
Y6α
Y4α
Monoisotopic mass:	1949.69 Da
Charge observed:		2-
Theoretical ion: 		m/z 973.85
Observed ion: 		m/z 973.97
Mass deviation:		0.12 Th
Retention time: 		53.3 min
2,4A6
H5N4G1a
D
Z2
1,3A3β
Y3β
Y5β
Y4β
Intens.
x105
1.0
943.94
790.85
0.8
0.6
1641.69
995.44
0.4
1540.59
851.41
883.91
1522.59
0.2
424.21
833.37
1583.63
1276.54
671.27
1114.47
1725.67
306.12
1872.80
553.13
1461.60
1403.65
689.35
1218.48
486.20
1813.74
0.0
400
600
800
1000
1200
1400
1600
1800
m/z
[M-60]2-
2,4A62-
Y6α1-
D1-
Y5β2-
C4α1-
C51-
B51-
Z5β2-
B4α1-
Y4α1-
Y4β1-
Z5α1-
1,3A3β1-
B61-
Y3α1-
Z3β1-
B3α1-
C2α1-
C3α1-
892.93
13

## Slide 14
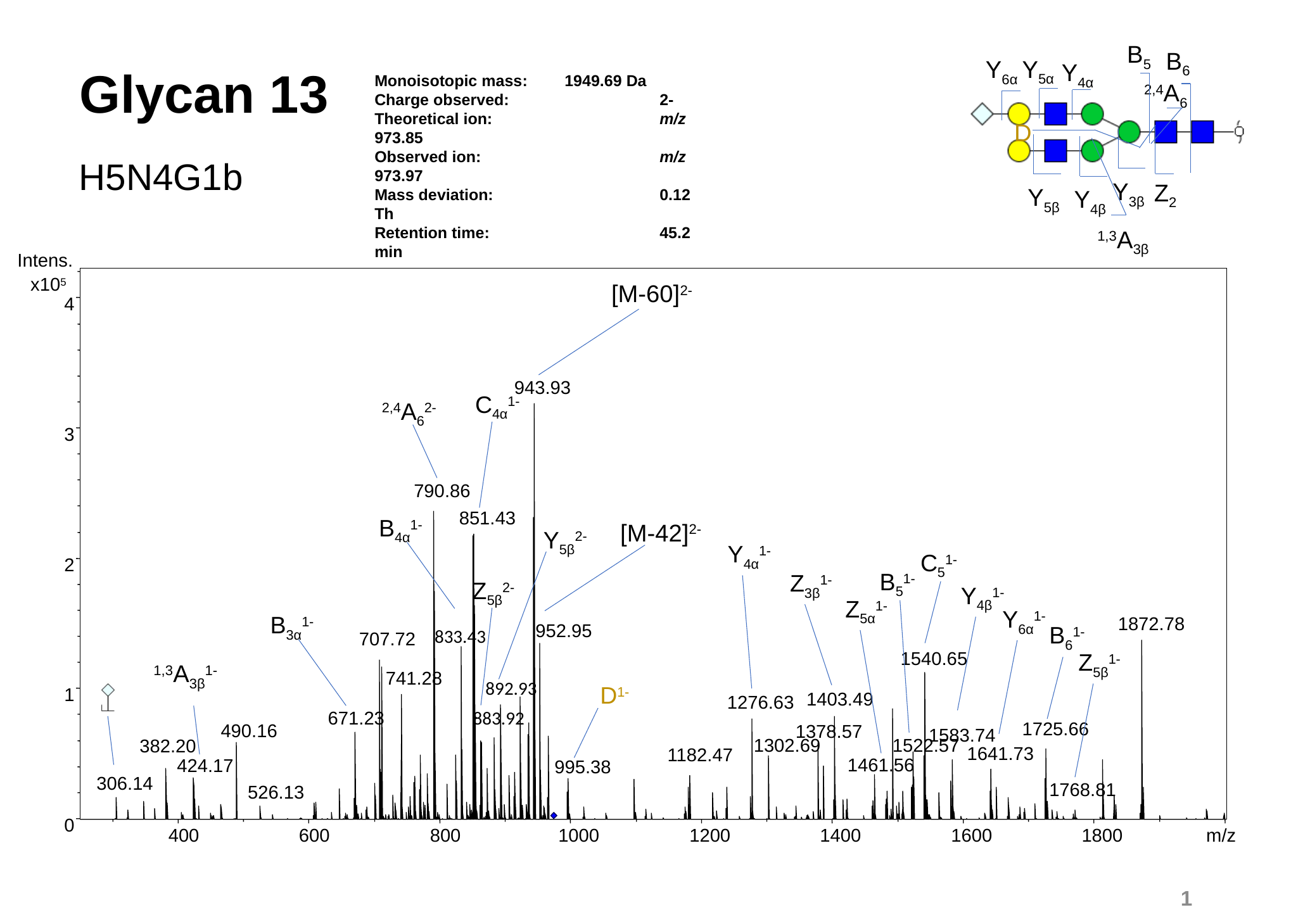

Glycan 13
B5
B6
Y5α
Y6α
Y4α
Monoisotopic mass:	1949.69 Da
Charge observed:		2-
Theoretical ion: 		m/z 973.85
Observed ion: 		m/z 973.97
Mass deviation:		0.12 Th
Retention time: 		45.2 min
2,4A6
H5N4G1b
D
Z2
1,3A3β
Y3β
Y5β
Y4β
Intens.
x105
4
943.93
3
790.86
851.43
2
1872.78
952.95
707.72
1540.65
741.28
1
1403.49
1276.63
671.23
1725.66
490.16
1378.57
1583.74
1302.69
1522.57
382.20
1641.73
1182.47
1461.56
424.17
995.38
306.14
1768.81
526.13
0
400
600
800
1000
1200
1400
1600
1800
m/z
[M-60]2-
C4α1-
2,4A62-
B4α1-
[M-42]2-
Y5β2-
Y4α1-
C51-
B51-
Z3β1-
Z5β2-
Y4β1-
Z5α1-
Y6α1-
B3α1-
B61-
833.43
Z5β1-
1,3A3β1-
D1-
892.93
883.92
14

## Slide 15
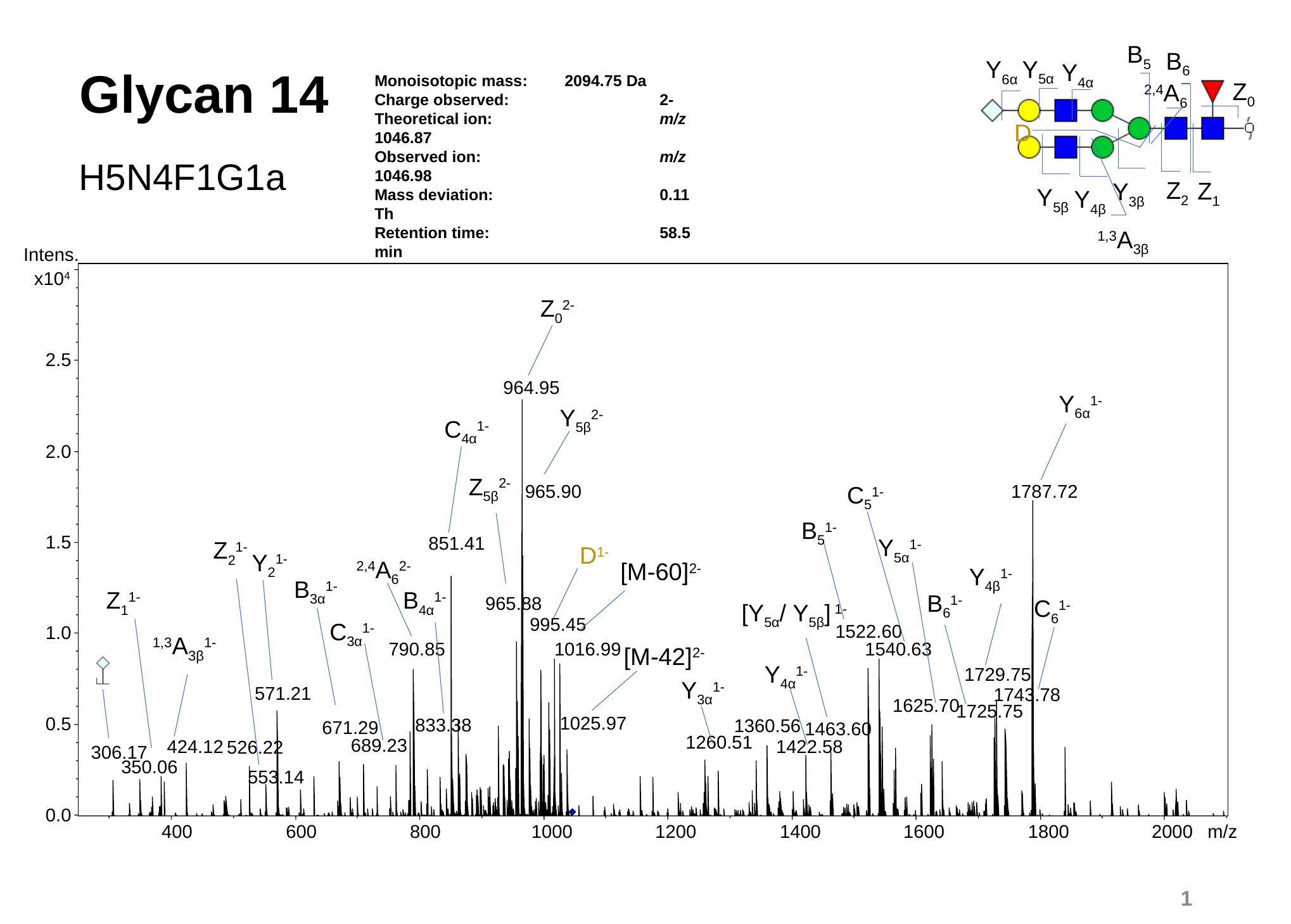

Glycan 14
B5
B6
Y5α
Y6α
Y4α
Monoisotopic mass:	2094.75 Da
Charge observed:		2-
Theoretical ion: 		m/z 1046.87
Observed ion: 		m/z 1046.98
Mass deviation:		0.11 Th
Retention time: 		58.5 min
Z0
2,4A6
H5N4F1G1a
D
Z2
Z1
1,3A3β
Y3β
Y5β
Y4β
Intens.
x104
2.5
964.95
2.0
1787.72
965.90
1.5
851.41
995.45
1522.60
1.0
790.85
1540.63
1016.99
571.21
1743.78
1625.70
1725.75
0.5
833.38
1360.56
671.29
1463.60
1260.51
689.23
424.12
1422.58
526.22
306.17
350.06
553.14
0.0
400
600
800
1000
1200
1400
1600
1800
2000
m/z
Z02-
Y6α1-
Y5β2-
C4α1-
Z5β2-
C51-
B51-
Y5α1-
Z21-
D1-
Y21-
2,4A62-
[M-60]2-
Y4β1-
B3α1-
B4α1-
Z11-
B61-
C61-
965.88
[Y5α/ Y5β] 1-
C3α1-
1,3A3β1-
[M-42]2-
Y4α1-
1729.75
Y3α1-
1025.97
15

## Slide 16
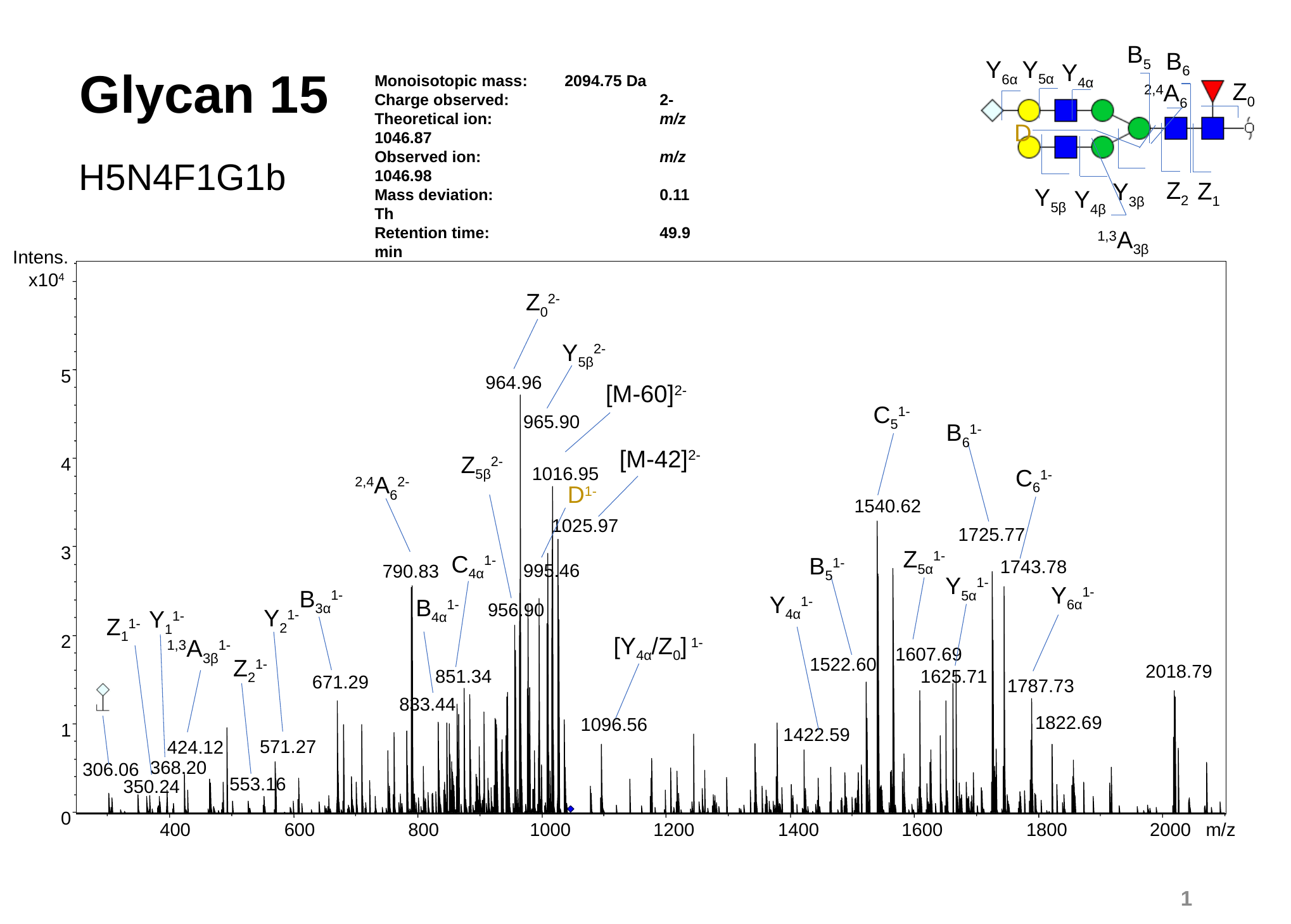

Glycan 15
B5
B6
Y5α
Y6α
Y4α
Monoisotopic mass:	2094.75 Da
Charge observed:		2-
Theoretical ion: 		m/z 1046.87
Observed ion: 		m/z 1046.98
Mass deviation:		0.11 Th
Retention time: 		49.9 min
Z0
2,4A6
H5N4F1G1b
D
Z2
Z1
1,3A3β
Y3β
Y5β
Y4β
Intens.
x104
5
964.96
4
1016.95
1540.62
1725.77
3
1743.78
995.46
790.83
956.90
2
1607.69
1522.60
2018.79
851.34
1625.71
671.29
1787.73
833.44
1822.69
1096.56
1
1422.59
571.27
424.12
368.20
306.06
553.16
350.24
0
400
600
800
1000
1200
1400
1600
1800
2000
m/z
Z02-
Y5β2-
[M-60]2-
C51-
965.90
B61-
[M-42]2-
Z5β2-
C61-
2,4A62-
D1-
1025.97
Z5α1-
C4α1-
B51-
Y5α1-
Y6α1-
B3α1-
Y4α1-
B4α1-
Y21-
Y11-
Z11-
[Y4α/Z0] 1-
1,3A3β1-
Z21-
16

## Slide 17
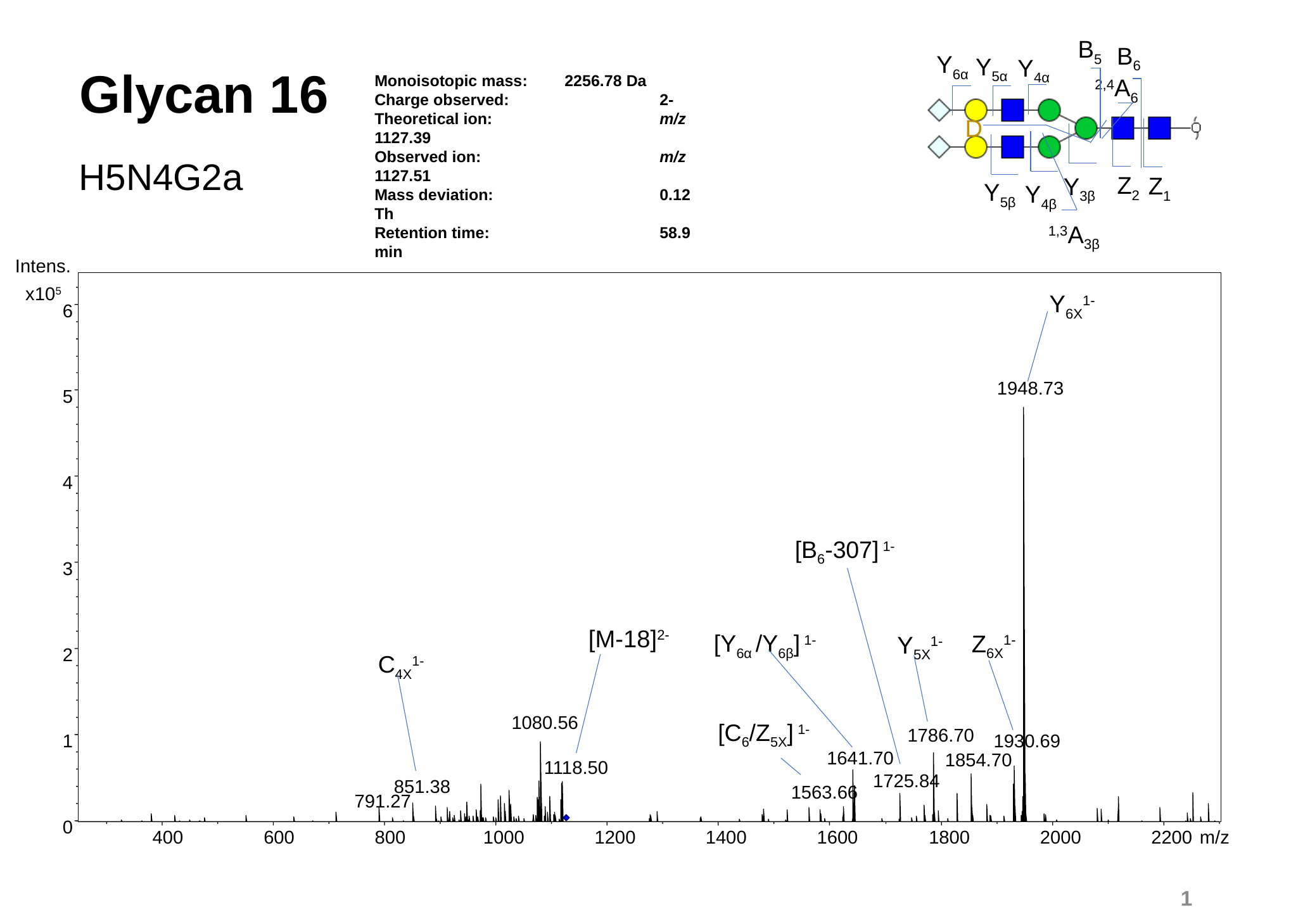

Glycan 16
B5
B6
Y6α
Y5α
Y4α
Monoisotopic mass:	2256.78 Da
Charge observed:		2-
Theoretical ion: 		m/z 1127.39
Observed ion: 		m/z 1127.51
Mass deviation:		0.12 Th
Retention time: 		58.9 min
2,4A6
H5N4G2a
D
Z2
Z1
1,3A3β
Y3β
Y5β
Y4β
Intens.
x105
6
1948.73
5
4
3
2
1080.56
1786.70
1
1930.69
1641.70
1854.70
1118.50
1725.84
851.38
1563.66
791.27
0
400
600
800
1000
1200
1400
1600
1800
2000
2200
m/z
Y6X1-
[B6-307] 1-
[M-18]2-
Z6X1-
[Y6α /Y6β] 1-
Y5X1-
C4X1-
[C6/Z5X] 1-
17

## Slide 18
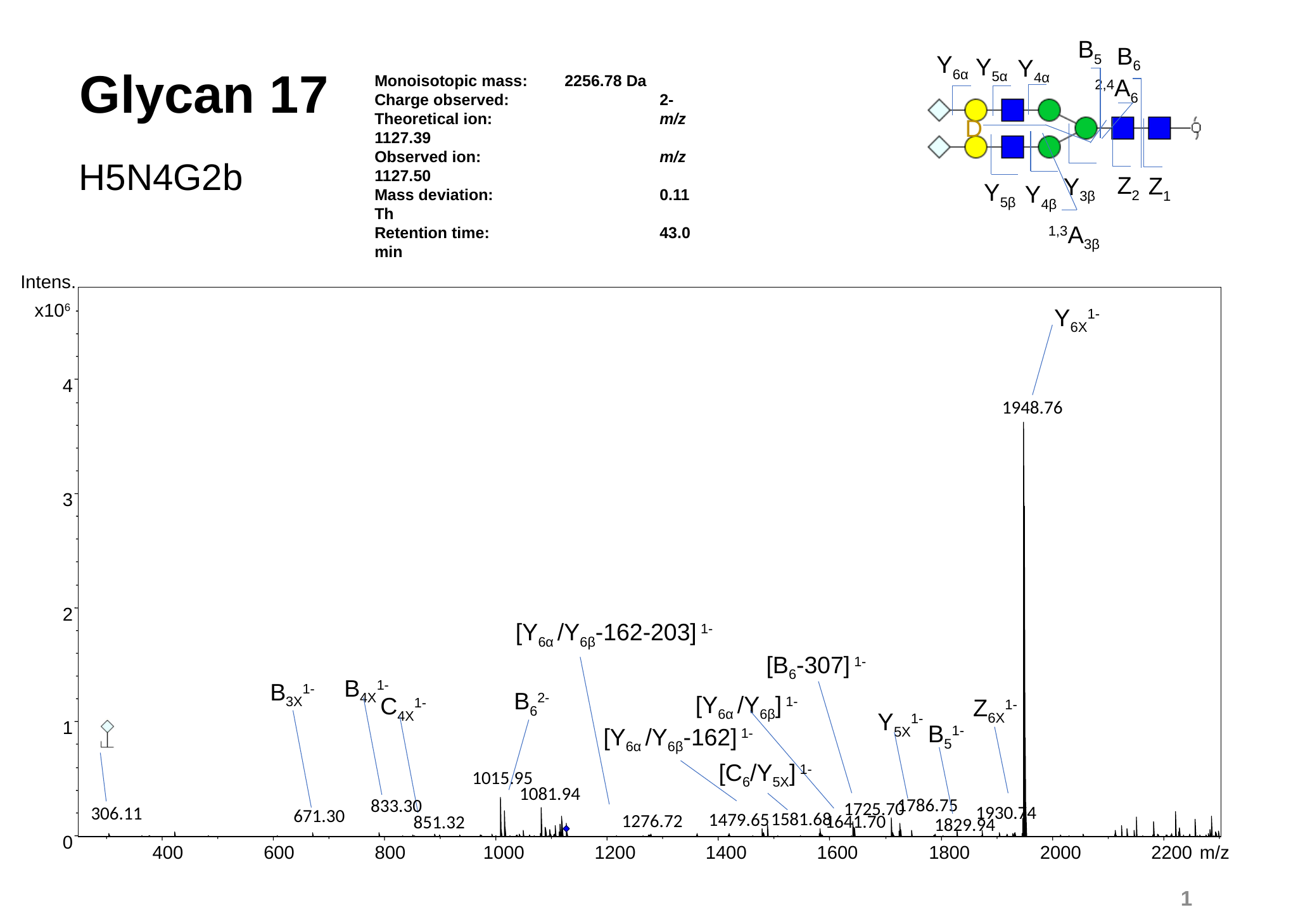

Glycan 17
B5
B6
Y6α
Y5α
Y4α
Monoisotopic mass:	2256.78 Da
Charge observed:		2-
Theoretical ion: 		m/z 1127.39
Observed ion: 		m/z 1127.50
Mass deviation:		0.11 Th
Retention time: 		43.0 min
2,4A6
H5N4G2b
D
Z2
Z1
1,3A3β
Y3β
Y5β
Y4β
Intens.
x106
4
1948.76
3
2
1
1015.95
1081.94
1786.75
1725.70
1930.74
1641.70
851.32
1829.94
0
400
600
800
1000
1200
1400
1600
1800
2000
2200
m/z
Y6X1-
[Y6α /Y6β-162-203] 1-
[B6-307] 1-
B4X1-
B3X1-
B62-
[Y6α /Y6β] 1-
C4X1-
Z6X1-
Y5X1-
B51-
[Y6α /Y6β-162] 1-
[C6/Y5X] 1-
833.30
306.11
671.30
1581.68
1479.65
1276.72
18

## Slide 19
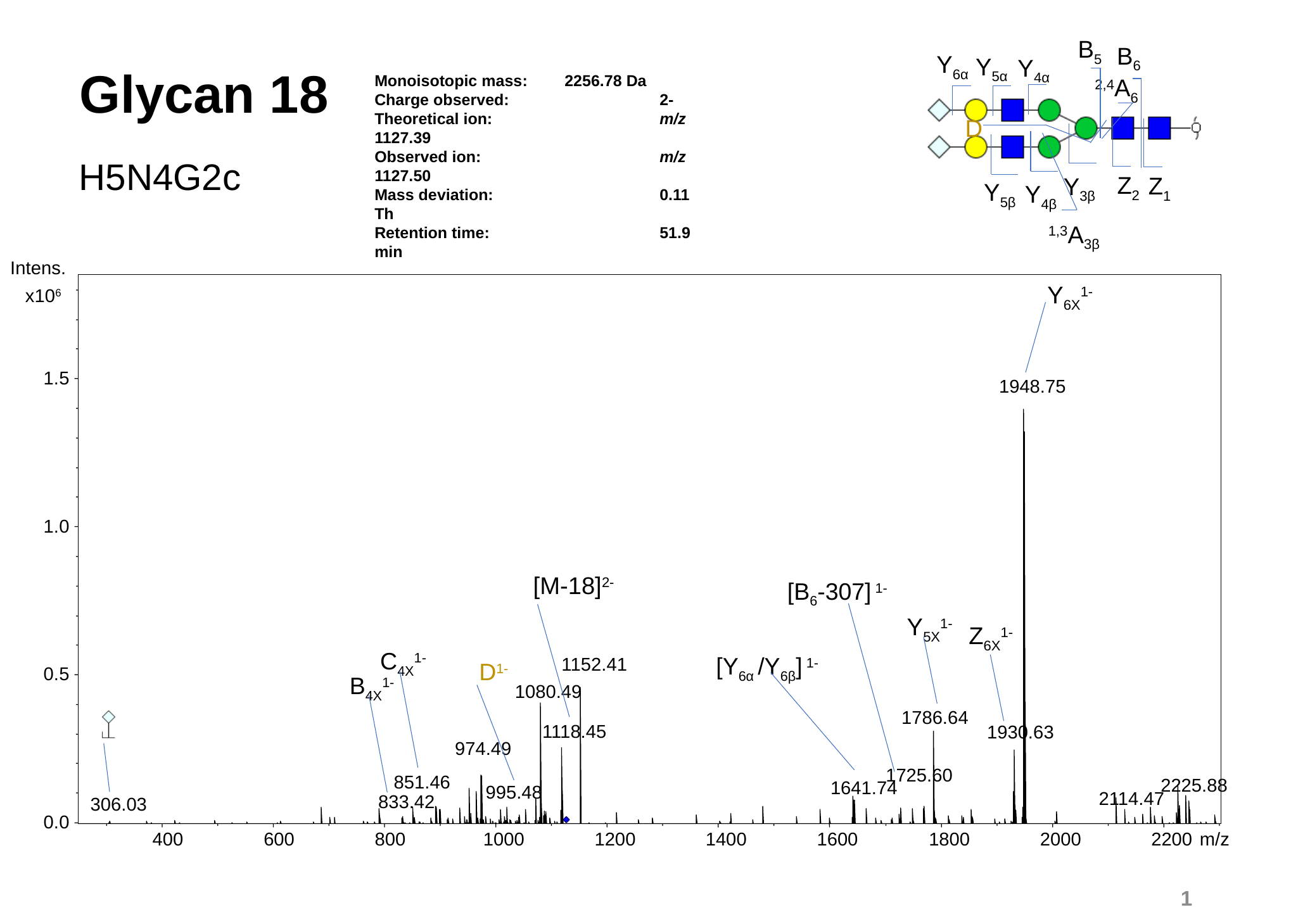

Glycan 18
B5
B6
Y6α
Y5α
Y4α
Monoisotopic mass:	2256.78 Da
Charge observed:		2-
Theoretical ion: 		m/z 1127.39
Observed ion: 		m/z 1127.50
Mass deviation:		0.11 Th
Retention time: 		51.9 min
2,4A6
H5N4G2c
D
Z2
Z1
1,3A3β
Y3β
Y5β
Y4β
Intens.
x106
1.5
1948.75
1.0
1152.41
0.5
1080.49
1786.64
1118.45
1930.63
974.49
1725.60
851.46
2225.88
1641.74
995.48
2114.47
833.42
306.03
0.0
400
600
800
1000
1200
1400
1600
1800
2000
2200
m/z
Y6X1-
[M-18]2-
[B6-307] 1-
Y5X1-
Z6X1-
C4X1-
[Y6α /Y6β] 1-
D1-
B4X1-
19

## Slide 20
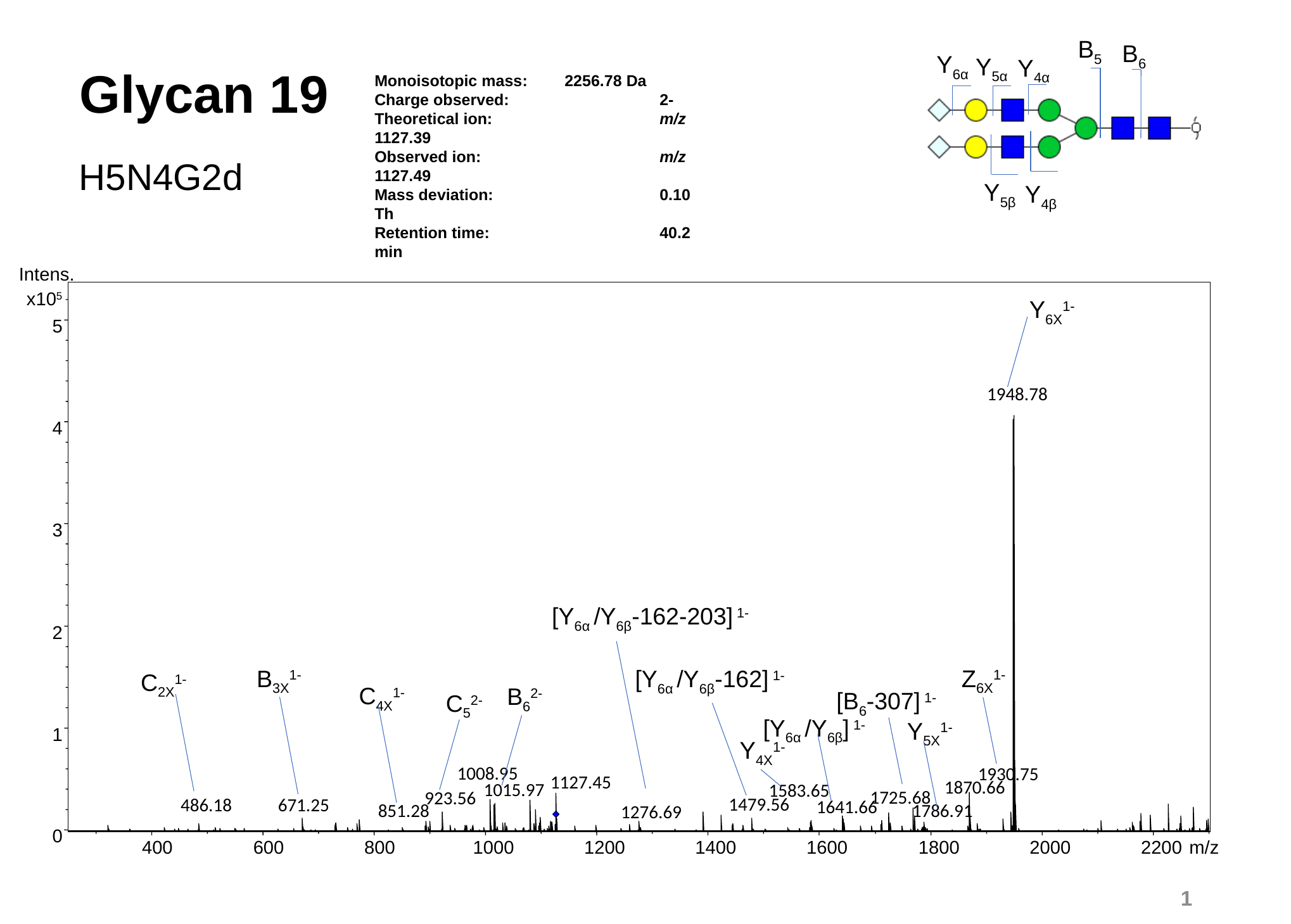

Glycan 19
B5
B6
Y6α
Y5α
Y4α
Monoisotopic mass:	2256.78 Da
Charge observed:		2-
Theoretical ion: 		m/z 1127.39
Observed ion: 		m/z 1127.49
Mass deviation:		0.10 Th
Retention time: 		40.2 min
H5N4G2d
Y5β
Y4β
Intens.
x105
5
1948.78
4
3
2
1
1008.95
1930.75
1127.45
1870.66
1015.97
1583.65
1725.68
923.56
1479.56
486.18
671.25
1641.66
851.28
1276.69
0
400
600
800
1000
1200
1400
1600
1800
2000
2200
m/z
Z02-
Y6X1-
[Y6α /Y6β-162-203] 1-
Z6X1-
B3X1-
[Y6α /Y6β-162] 1-
C2X1-
C4X1-
B62-
[B6-307] 1-
C52-
[Y6α /Y6β] 1-
Y5X1-
Y4X1-
1786.91
20

## Slide 21
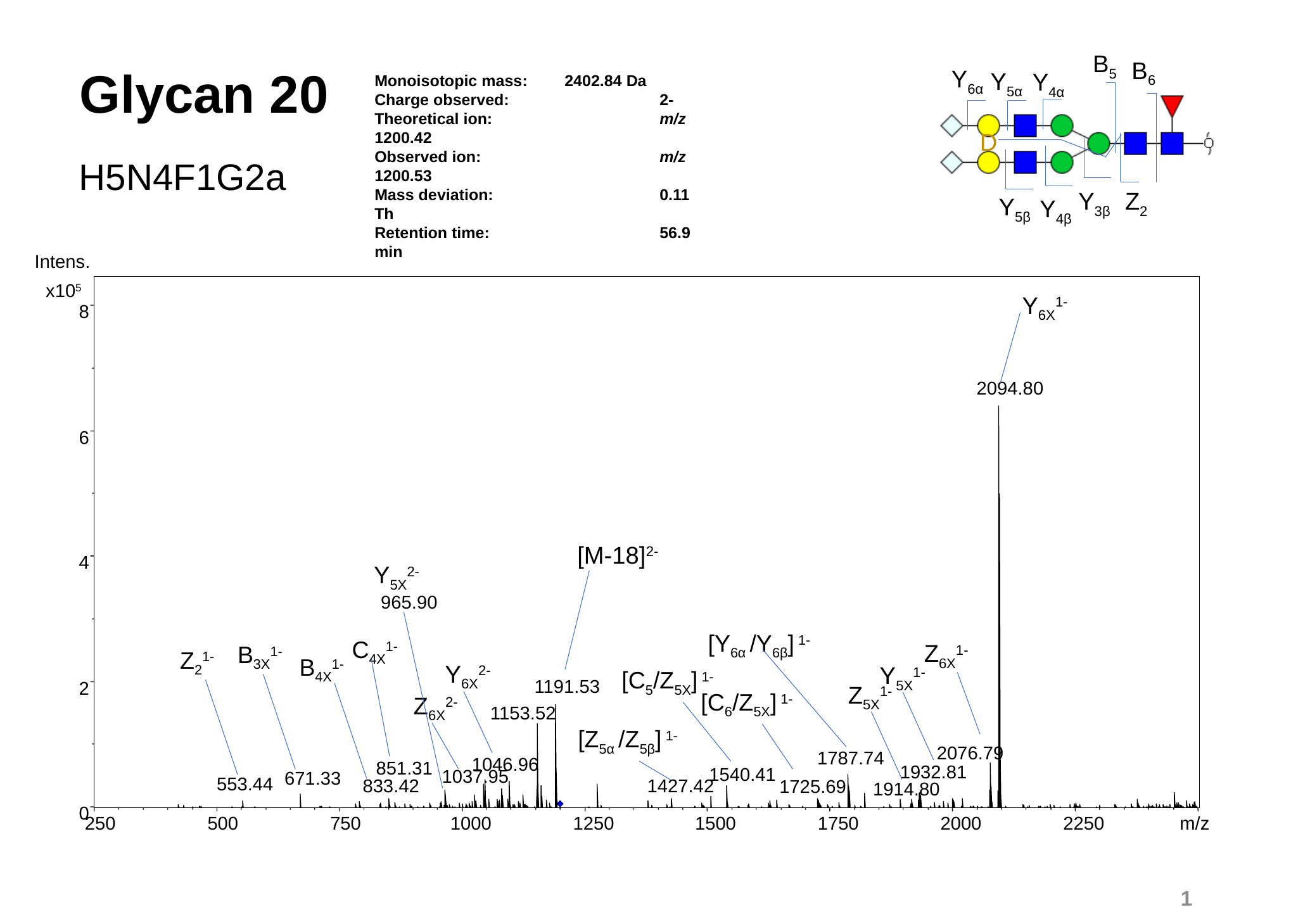

Glycan 20
B5
B6
Y6α
Y5α
Y4α
Monoisotopic mass:	2402.84 Da
Charge observed:		2-
Theoretical ion: 		m/z 1200.42
Observed ion: 		m/z 1200.53
Mass deviation:		0.11 Th
Retention time: 		56.9 min
H5N4F1G2a
D
Z2
Y3β
Y5β
Y4β
Intens.
x105
8
2094.80
6
4
1191.53
2
1153.52
2076.79
1787.74
1046.96
851.31
1932.81
1540.41
1037.95
671.33
833.42
1427.42
1725.69
1914.80
0
250
500
750
1000
1250
1500
1750
2000
2250
m/z
Y6X1-
[M-18]2-
Y5X2-
965.90
[Y6α /Y6β] 1-
C4X1-
Z6X1-
B3X1-
Z21-
B4X1-
Y6X2-
Y5X1-
[C5/Z5X] 1-
Z5X1-
[C6/Z5X] 1-
Z6X2-
[Z5α /Z5β] 1-
553.44
21

## Slide 22
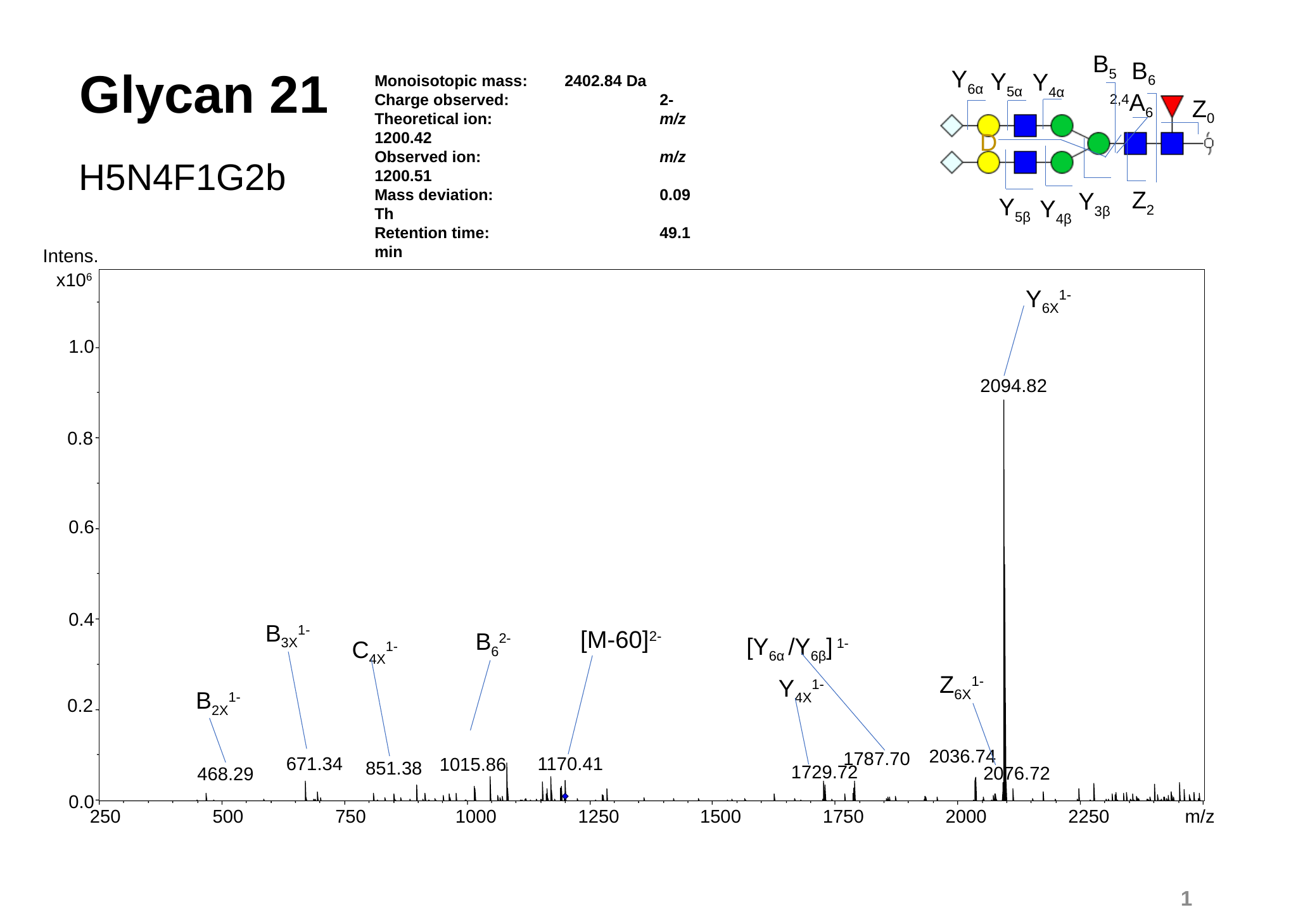

Glycan 21
B5
B6
Y6α
Y5α
Y4α
Monoisotopic mass:	2402.84 Da
Charge observed:		2-
Theoretical ion: 		m/z 1200.42
Observed ion: 		m/z 1200.51
Mass deviation:		0.09 Th
Retention time: 		49.1 min
2,4A6
Z0
H5N4F1G2b
D
Z2
Y3β
Y5β
Y4β
Intens.
x106
1.0
2094.82
0.8
0.6
0.4
0.2
2036.74
1787.70
671.34
1170.41
1015.86
1729.72
2076.72
468.29
0.0
250
500
750
1000
1250
1500
1750
2000
2250
m/z
Y6X1-
B3X1-
[M-60]2-
B62-
[Y6α /Y6β] 1-
C4X1-
Z6X1-
Y4X1-
B2X1-
851.38
22

## Slide 23
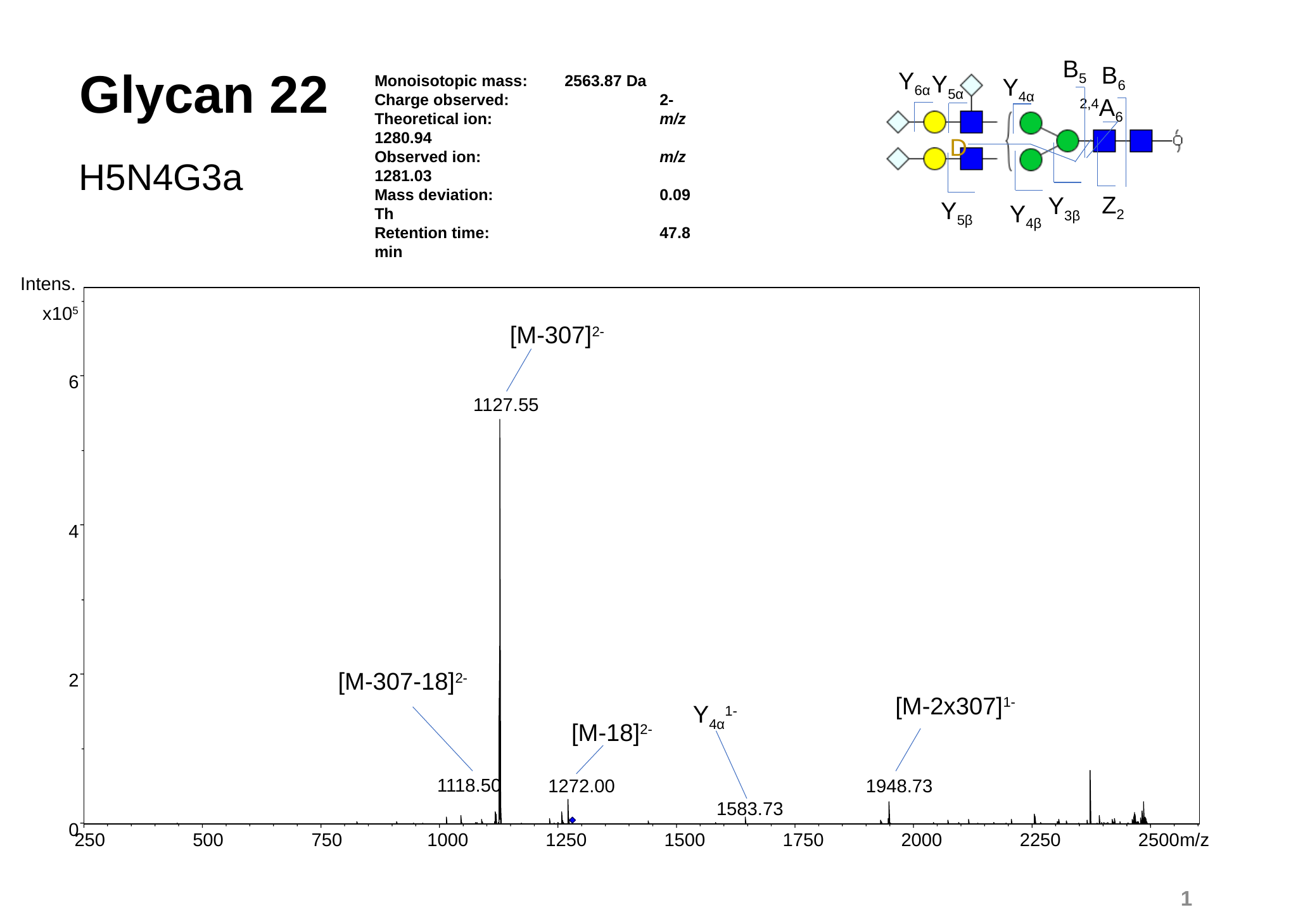

Glycan 22
B5
B6
Y6α
Y5α
Monoisotopic mass:	2563.87 Da
Charge observed:		2-
Theoretical ion: 		m/z 1280.94
Observed ion: 		m/z 1281.03
Mass deviation:		0.09 Th
Retention time: 		47.8 min
Y4α
2,4A6
H5N4G3a
D
Z2
Y3β
Y5β
Y4β
Intens.
x105
6
1127.55
4
2
1118.50
1272.00
1948.73
1583.73
0
250
500
750
1000
1250
1500
1750
2000
2250
2500
m/z
[M-307]2-
[M-307-18]2-
[M-2x307]1-
Y4α1-
[M-18]2-
23

## Slide 24
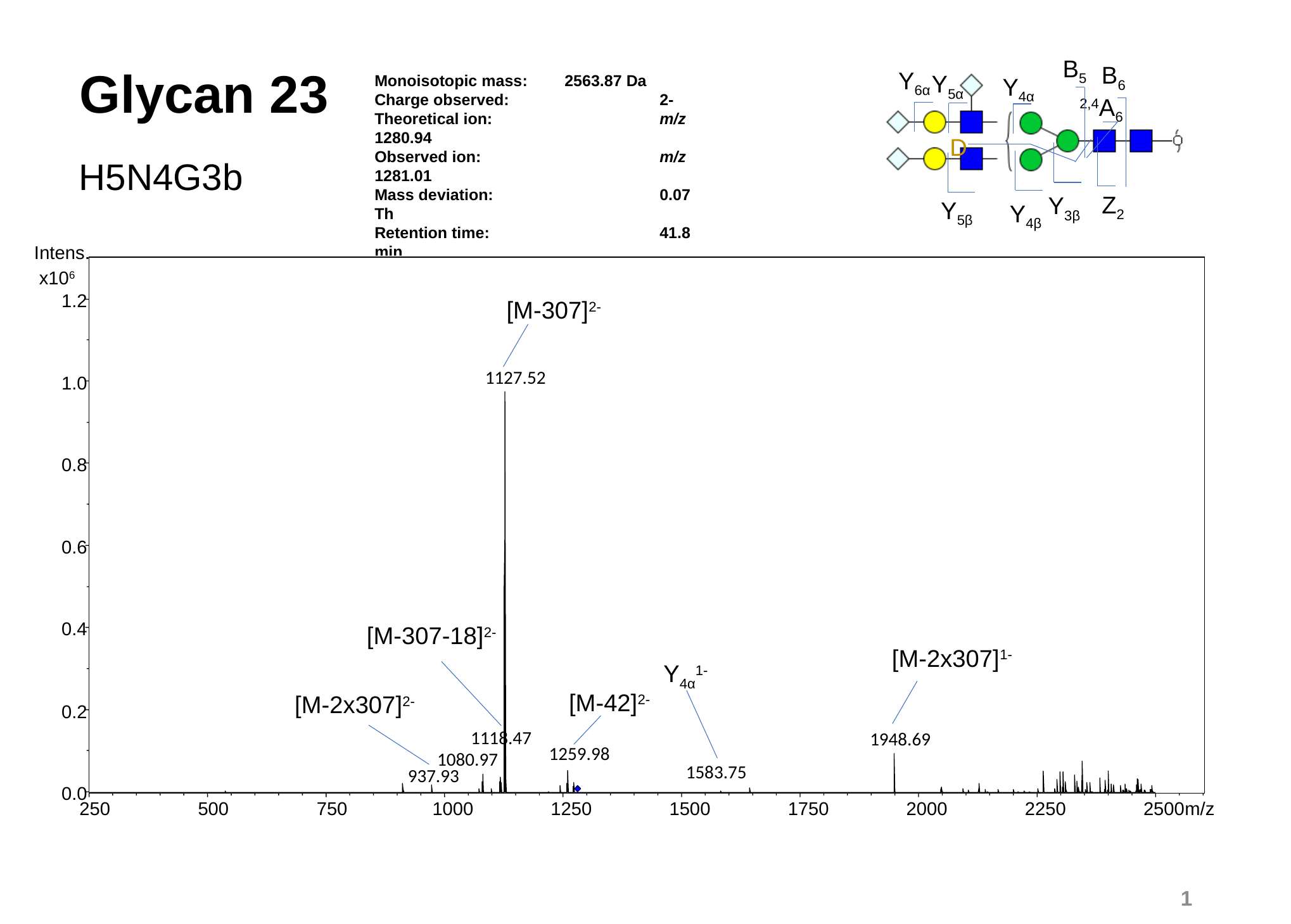

Glycan 23
B5
B6
Y6α
Y5α
Monoisotopic mass:	2563.87 Da
Charge observed:		2-
Theoretical ion: 		m/z 1280.94
Observed ion: 		m/z 1281.01
Mass deviation:		0.07 Th
Retention time: 		41.8 min
Y4α
2,4A6
H5N4G3b
D
Z2
Y3β
Y5β
Y4β
Intens.
x106
1.2
1127.52
1.0
0.8
0.6
0.4
0.2
1118.47
1948.69
1259.98
1080.97
1583.75
937.93
0.0
250
500
750
1000
1250
1500
1750
2000
2250
2500
m/z
[M-307]2-
[M-307-18]2-
[M-2x307]1-
Y4α1-
[M-42]2-
[M-2x307]2-
24

## Slide 25
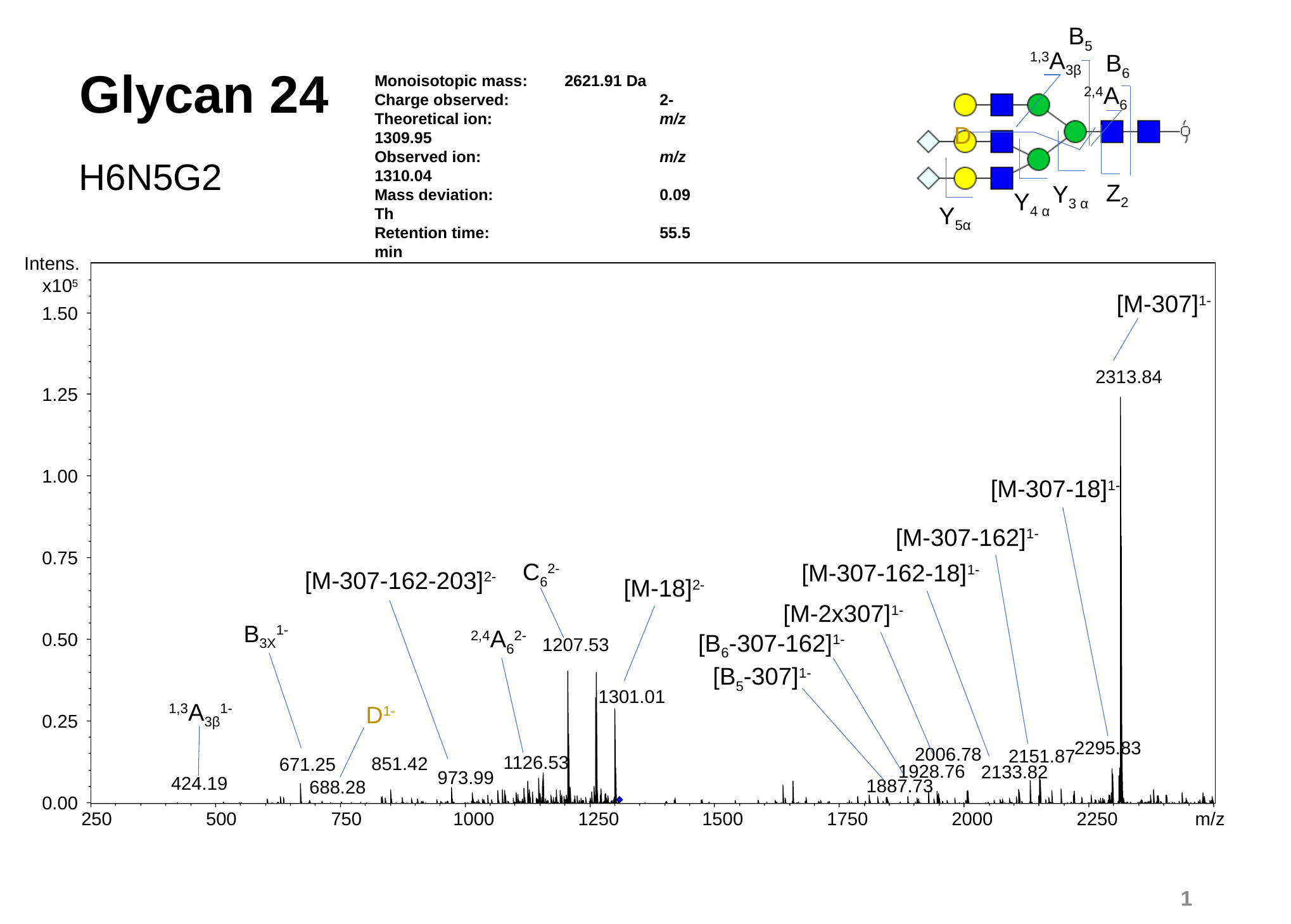

B5
Glycan 24
1,3A3β
B6
Monoisotopic mass:	2621.91 Da
Charge observed:		2-
Theoretical ion: 		m/z 1309.95
Observed ion: 		m/z 1310.04
Mass deviation:		0.09 Th
Retention time: 		55.5 min
2,4A6
H6N5G2
D
Z2
Y3 α
Y4 α
Y5α
Intens.
x105
1.50
2313.84
1.25
1.00
0.75
0.50
1207.53
1301.01
0.25
2295.83
2006.78
2151.87
1126.53
851.42
671.25
1928.76
2133.82
973.99
424.19
1887.73
688.28
0.00
250
500
750
1000
1250
1500
1750
2000
2250
m/z
[M-307]1-
[M-307-18]1-
[M-307-162]1-
C62-
[M-307-162-18]1-
[M-307-162-203]2-
[M-18]2-
[M-2x307]1-
B3X1-
2,4A62-
[B6-307-162]1-
[B5-307]1-
1,3A3β1-
D1-
25

## Slide 26
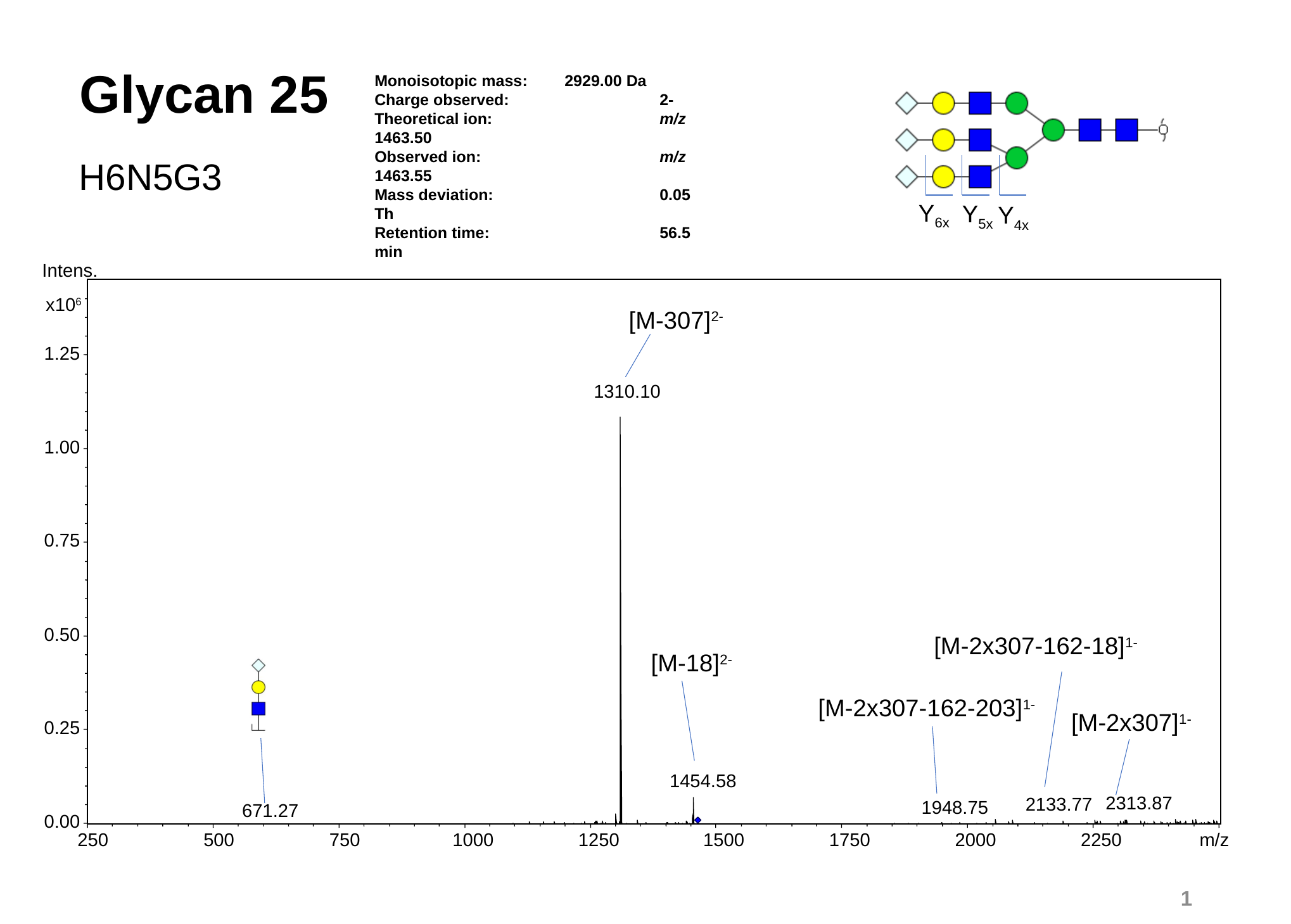

Glycan 25
Monoisotopic mass:	2929.00 Da
Charge observed:		2-
Theoretical ion: 		m/z 1463.50
Observed ion: 		m/z 1463.55
Mass deviation:		0.05 Th
Retention time: 		56.5 min
H6N5G3
Y6x
Y5x
Y4x
Intens.
x106
1.25
1310.10
1.00
0.75
0.50
0.25
1454.58
671.27
0.00
250
500
750
1000
1250
1500
1750
2000
2250
m/z
[M-307]2-
[M-2x307-162-18]1-
[M-18]2-
[M-2x307-162-203]1-
[M-2x307]1-
2313.87
2133.77
1948.75
26

## Slide 27
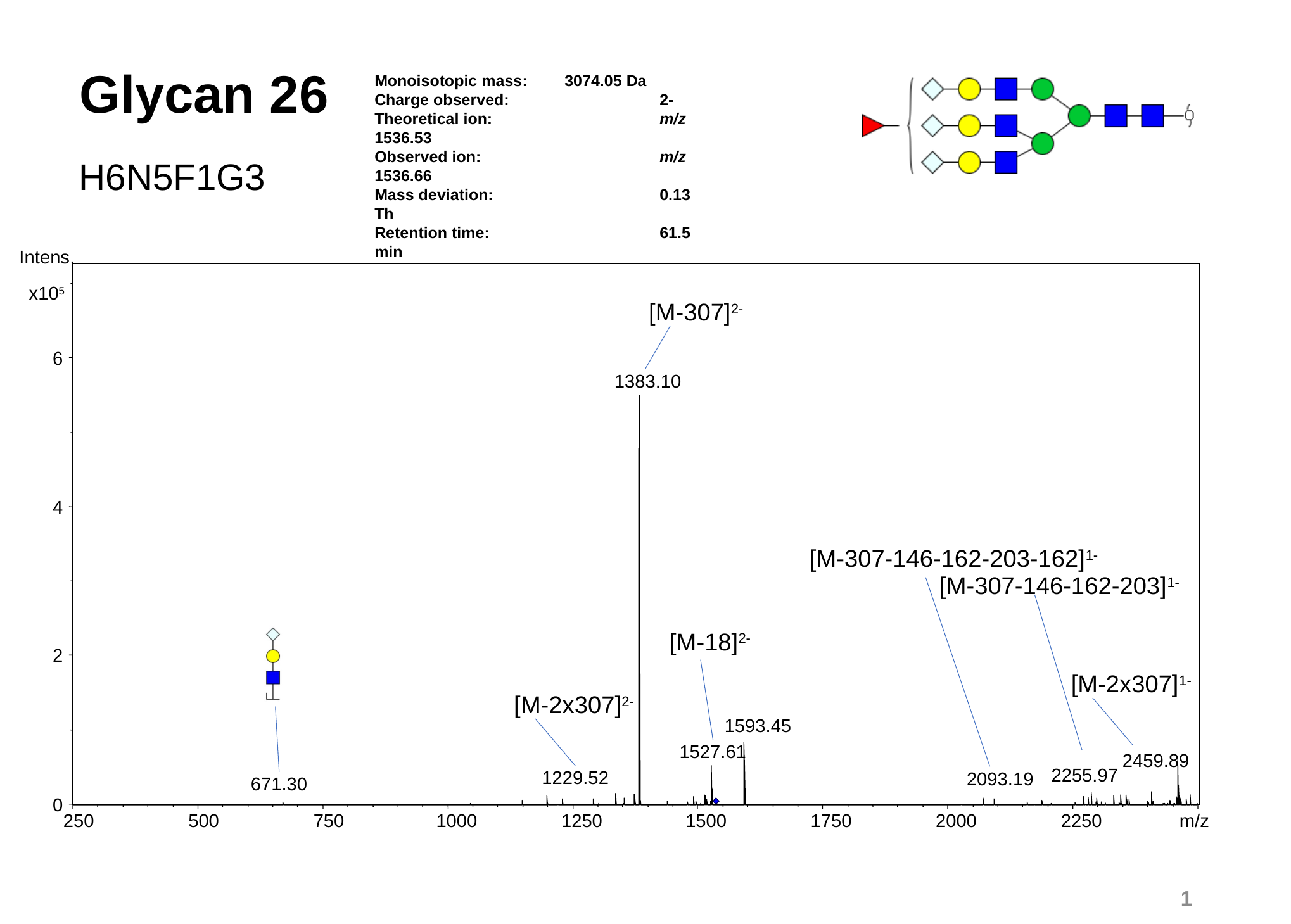

Glycan 26
Monoisotopic mass:	3074.05 Da
Charge observed:		2-
Theoretical ion: 		m/z 1536.53
Observed ion: 		m/z 1536.66
Mass deviation:		0.13 Th
Retention time: 		61.5 min
H6N5F1G3
Intens.
x105
6
1383.10
4
2
1593.45
1527.61
1229.52
2093.19
671.30
0
250
500
750
1000
1250
1500
1750
2000
2250
m/z
[M-307]2-
[M-307-146-162-203-162]1-
[M-307-146-162-203]1-
[M-18]2-
[M-2x307]1-
[M-2x307]2-
2459.89
2255.97
27

## Slide 28
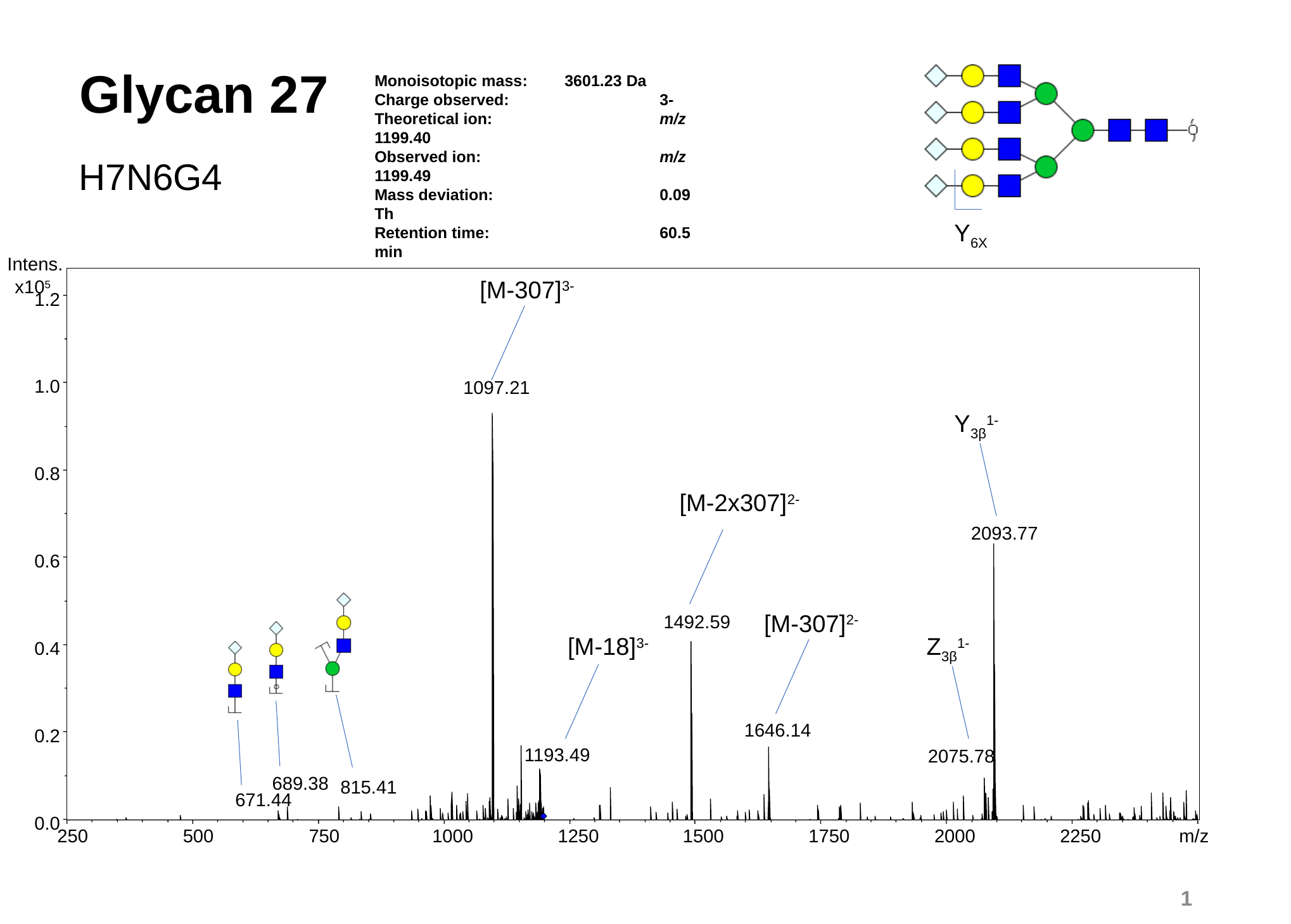

Glycan 27
Monoisotopic mass:	3601.23 Da
Charge observed:		3-
Theoretical ion: 		m/z 1199.40
Observed ion: 		m/z 1199.49
Mass deviation:		0.09 Th
Retention time: 		60.5 min
H7N6G4
Y6X
Intens.
x105
1.2
1.0
1097.21
0.8
2093.77
0.6
1492.59
0.4
1646.14
0.2
1193.49
2075.78
689.38
815.41
0.0
250
500
750
1000
1250
1500
1750
2000
2250
m/z
[M-307]3-
Y3β1-
[M-2x307]2-
[M-307]2-
[M-18]3-
Z3β1-
671.44
28
