# Supplementary material for: Impact of Asialoglycoprotein Receptor and Mannose Receptor Deficiency on Murine Plasma N-glycome Profiles
Source: Mol Cell Proteomics. 2023 Jul 4;22(9):100615. doi: 10.1016/j.mcpro.2023.100615 (PMC10462831; doi:10.1016/j.mcpro.2023.100615)
Supplement: Supplemental Figures S1–S6 and Tables S1–S21 [file mmc2.pdf]

Supplementary data

Impact of asialoglycoprotein receptor and mannose receptor deficency on murine plasma N-glycome profiles

M. Svecla[1,2], J. Nour[1], M.R. Bladergroen[2], S. Nicolardi[2], T. Zhang[2], G. Beretta[3], M. Wuhrer[2], G.D. Norata[1,4], D. Falck[2]

[1] Department of Pharmacological and Biomolecular Sciences, Università degli Studi di Milano, Milan, Italy; [2] Center for Proteomics and Metabolomics, Leiden University Medical Center, Leiden, The Netherlands; [3]Department of Environmental Science and Policy, Università degli Studi di Milano, Milan, Italy; [4]Centro SISA per lo studio dell'Aterosclerosi, Ospedale Bassini, Cinisello Balsamo, Italy

Corresponding author: David Falck, e-mail: D.Falck@lumc.nl

Supplemental content (this file):

Supplementary Figure S1 – Confirmation of genotype in ASGR1 and MRC1 deficient mice.

Supplementary Figure S2 – Technical variability.

Supplementary Figure S3 – Glycosylation traits in ASGR1 deficient mice.

Supplementary Figure S4 – Glycan abundances in ASGR1 deficient mice.

Supplementary Figure S5 – Glycosylation traits in MRC1 deficient mice.

Supplementary Figure S6 – Glycan abundances in MRC1 deficient mice.

Supplemental content (excel file):

Supplementary Table S1 – Calibrants list for internal calibration by MassyTools (version 2.0.0). All masses are shown as $[M+Na]^+$. The calibration window was set to 0.4, minimum signal-to-noise ratio for calibrants 9, minimum number of calibrants throughout entire spectrum 5 and the extraction width was set to 0.075.

Supplementary Table S2 – The calculation for glycosylation traits based on the identified glycans described in supplementary Table S1.

Supplementary Table S3– N-glycan monosaccharide composition and the exact mass of their $[M+Na]^+$ in TPNG.

Supplementary Table S4 – Abundance (LFQ based) of 23 plasma glycoproteins of WT (n=4) and ASGR1$^{-/-}$ (n=4) mice. The proteins are sorted by decreasing confidence based on the number of peptides detected.

Supplementary Table S5 – Abundance (LFQ based) of 23 plasma glycoproteins of WT (n=4) and MRC1$^{-/-}$ (n=4) mice. The proteins are sorted by decreasing confidence based on the number of peptides detected.

Supplementary Table S6-S13 – Accession, Score, Coverage, Peptides, PSM, Spectra, Cluster, Protein name, Decoy and FDR q-value for each protein assigned in WT and ASGR1$^{-/-}$ mice.

Supplementary Table S14-S21 – Accession, Score, Coverage, Peptides, PSM, Spectra, Cluster, Protein name, Decoy and FDR q-value for each protein assigned in WT and MRC1$^{-/-}$ mice.

Legend: H = hexose; N = *N*-Acetylhexosamine; F = fucose; E = α2,6-linked *N*-Acetylneuraminic acid; L= α2,3-linked *N*-Acetylneuraminic acid; Ge = α2,6-linked *N*-Glycolylneuraminic acid; Gl= α2,3-linked *N*-Glycolylneuraminic acid; Ac = acetyl group

Supplementary Figure S1

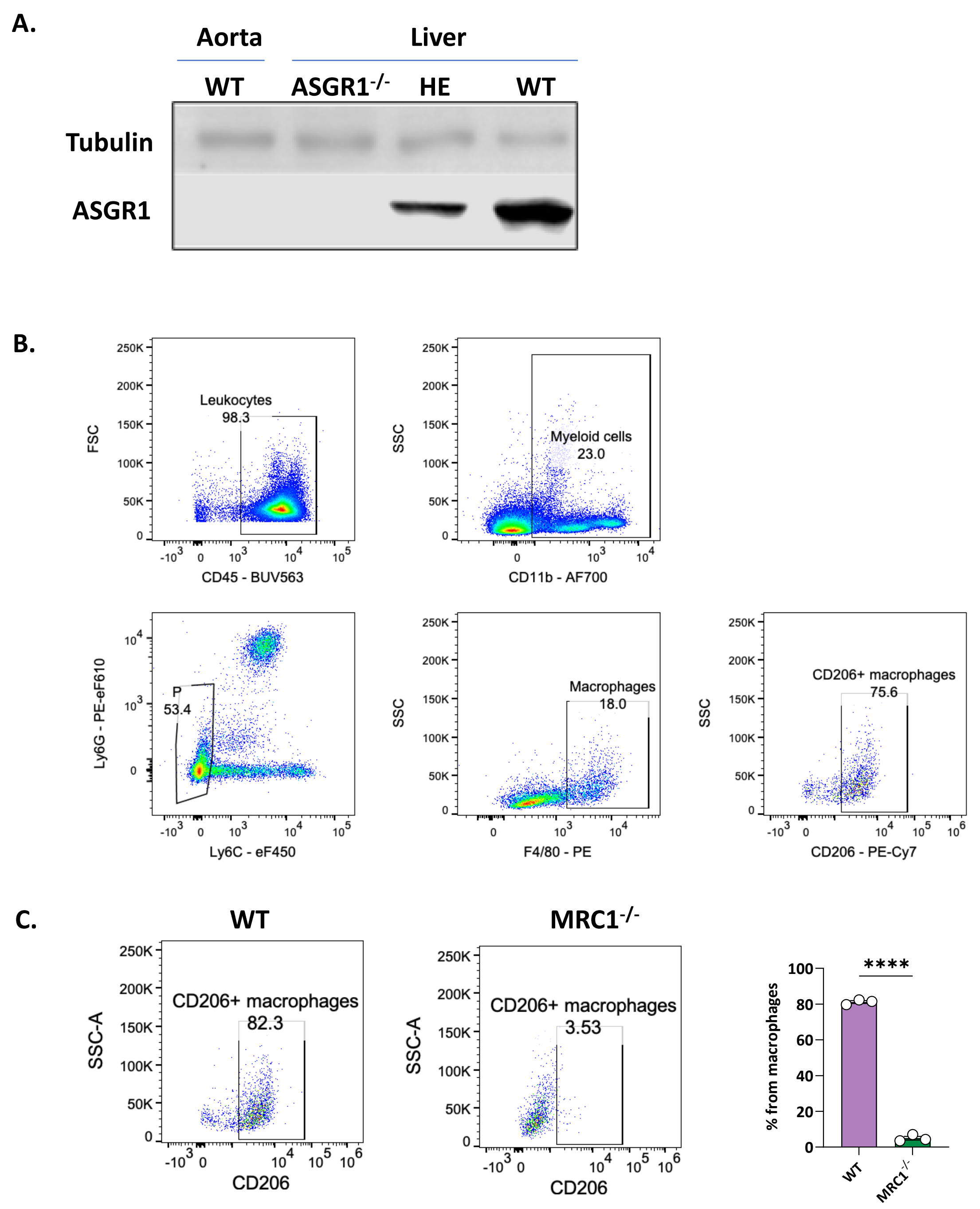

Supplementary Figure S1 – Confirmation of genotype in ASGR1 and MRC1 deficient mice. A) A representative western blot for ASGR1 on liver and aorta lysate. B) Flow cytometry gating strategy for identifying MRC1-positive ($CD206^+$) macrophages in the spleens of WT and $MRC1^{-/-}$ mice. C) MRC1-positive ($CD206^+$) macrophages and the percentage of $CD206^+$ cells in the spleens of WT (n=3) and $MRC1^{-/-}$ mice.

Supplementary Figure S2

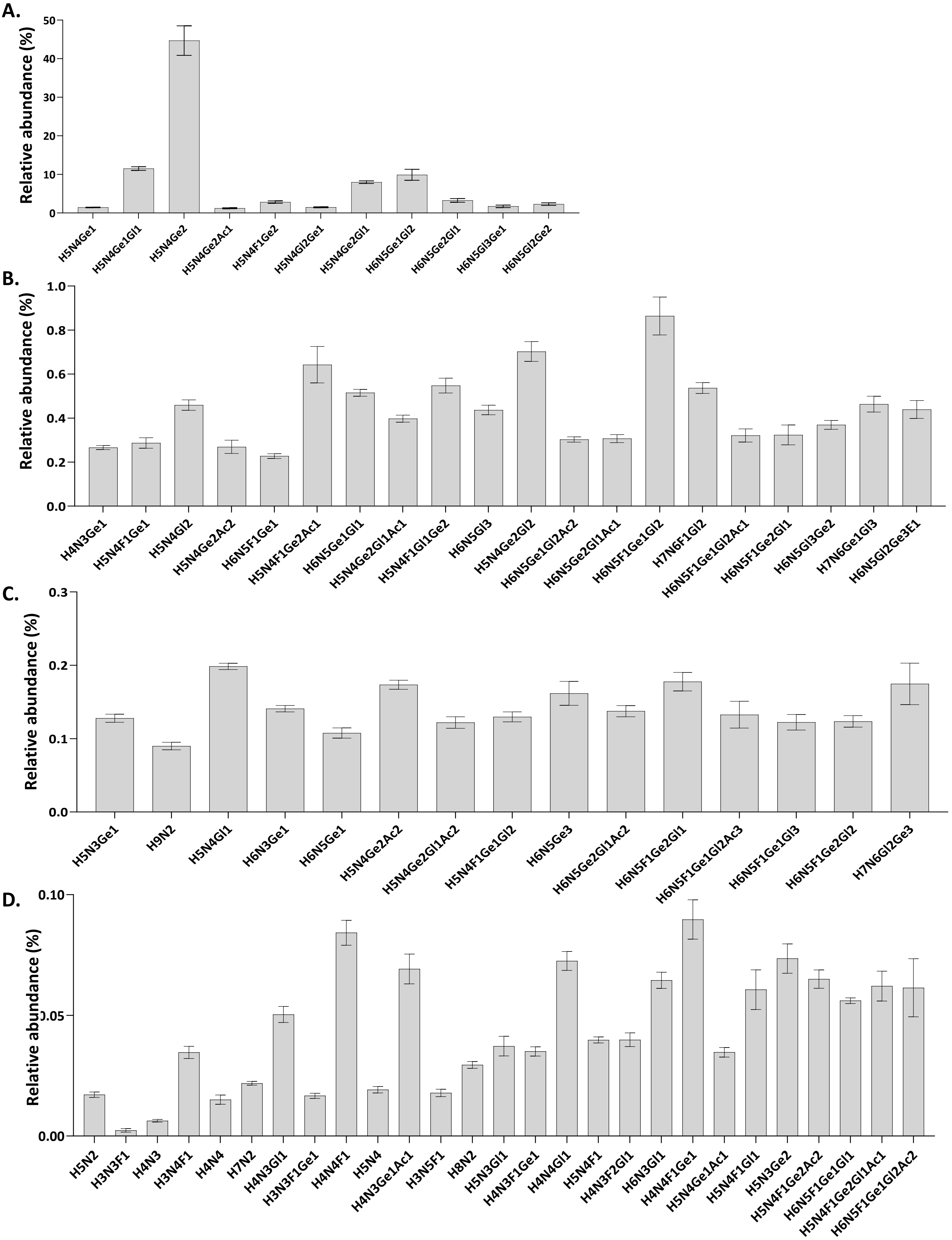

Supplementary Figure S2 – Technical variability. High precision over all measurements is exemplified by repeated measurements of a technical standard (pool of mice plasma). Glycans are sorted by relative abundance into the different panels. A) >1%, B) 1% to 0.3%, C) 0.3% to 0.1%, D) <0.1%. In each panel, glycan abundances are depicted in ascending order of mass. The error bars show standard deviation of the mean (n=6). H = hexose; N = *N*-Acetylhexosamine; F = fucose; E or L = *N*-Acetylneuraminic acid for α2,6- and α2,3-linked variants, respectively; Ge or Gl= *N*-Glycolylneuraminic acid for α2,6- and α2,3-linked variants, respectively; Ac = acetyl group.

Supplementary Figure S3

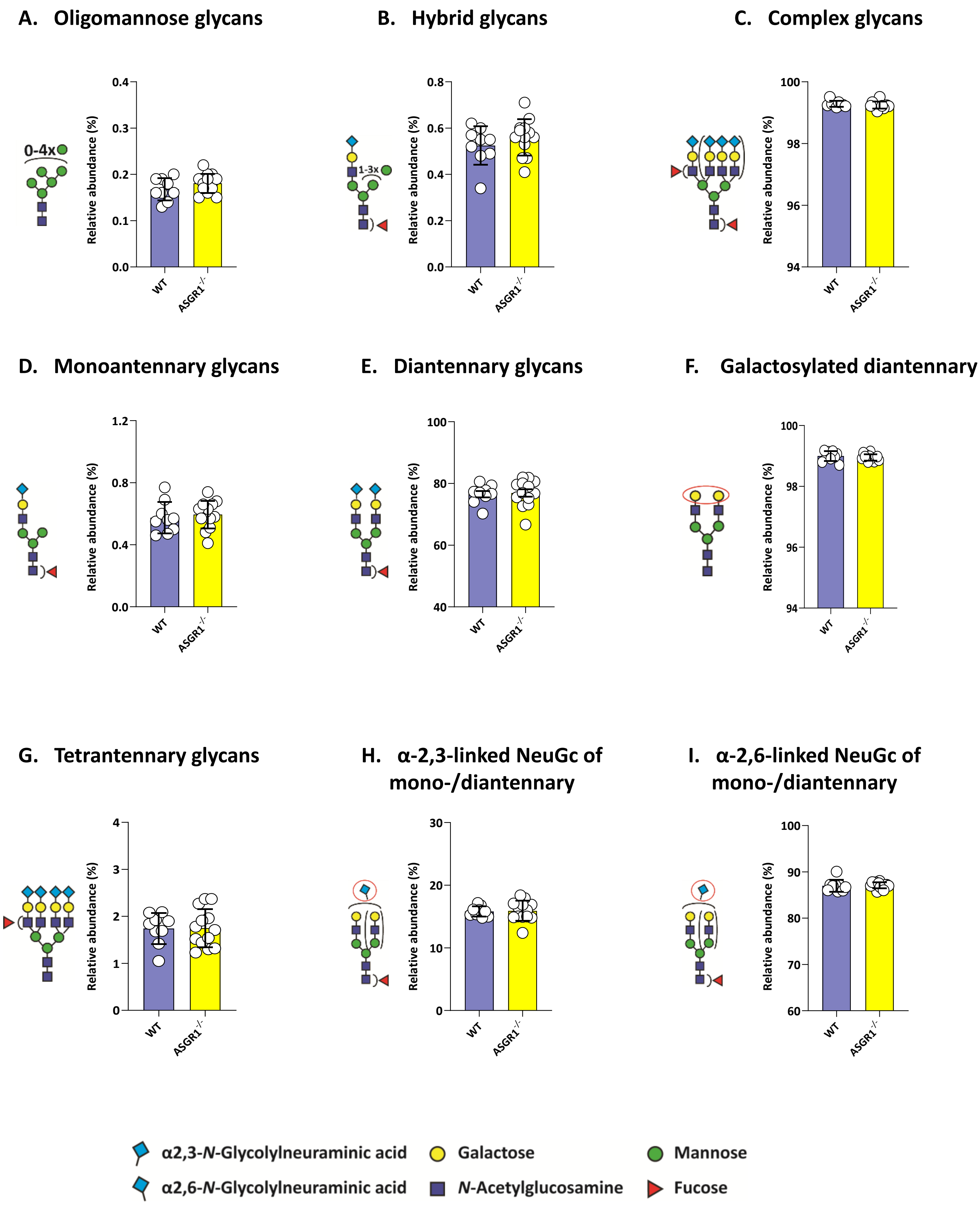

Supplementary Figure S3 – Glycosylation traits in ASGR1 deficient mice. A) Oligomannose, B) Hybrid glycans, C) Complex glycans, D) Monoantennary glycans, E) Diantennary glycans, F) Galactosylated diantennary glycans G) Tetrantennary glycans, H) α2,3-linked *N*-Glycolylneuraminic acid of mono-/diantennary glycans, I) α2,6-linked *N*-Glycolylneuraminic acid of mono-/diantennary glycans. Data are shown as mean ± SD of WT (n=9) and ASGR1$^{-/-}$ (n=12).

Supplementary Figure S4

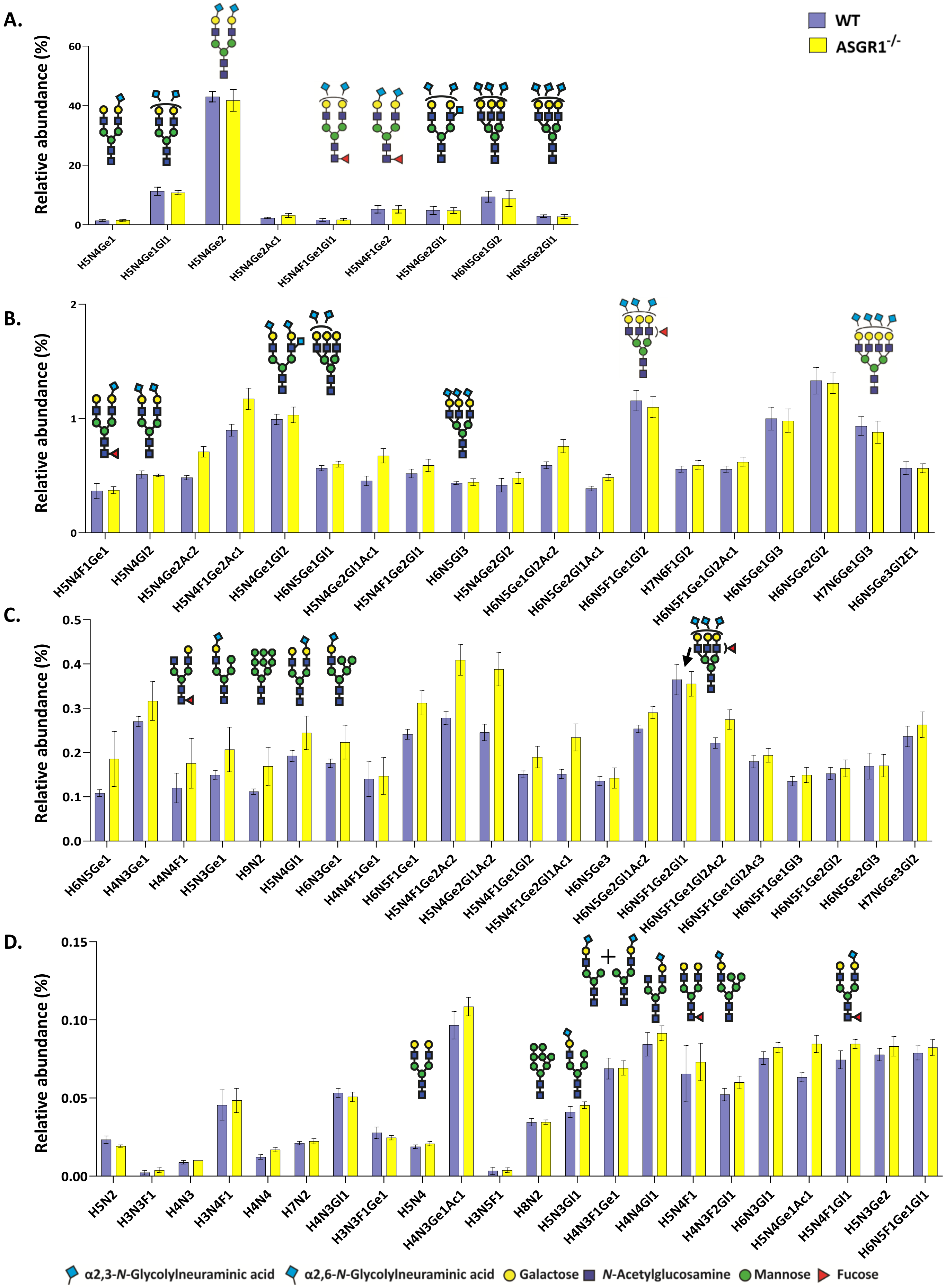

Supplementary Figure S4 – Glycan abundances in ASGR1 deficient mice. Glycans are sorted by relative abundance into the different panels. A) >1.5%, B) 1.5% to 0.4%, C) 0.4% to 0.1%, D) <0.1%. In each panel, glycan abundances are depicted in ascending order of mass. Data are shown as mean ± SEM of the WT (n=9) and ASGR1$^{-/-}$ (n=12). H = hexose; N = *N*-Acetylhexosamine; F = fucose; E or L = *N*-Acetylneuraminic acid for α2,6- and α2,3-linked variants, respectively; Ge or Gl= *N*-Glycolylneuraminic acid for α2,6- and α2,3-linked variants, respectively; Ac = acetyl group.

Supplementary Figure S5

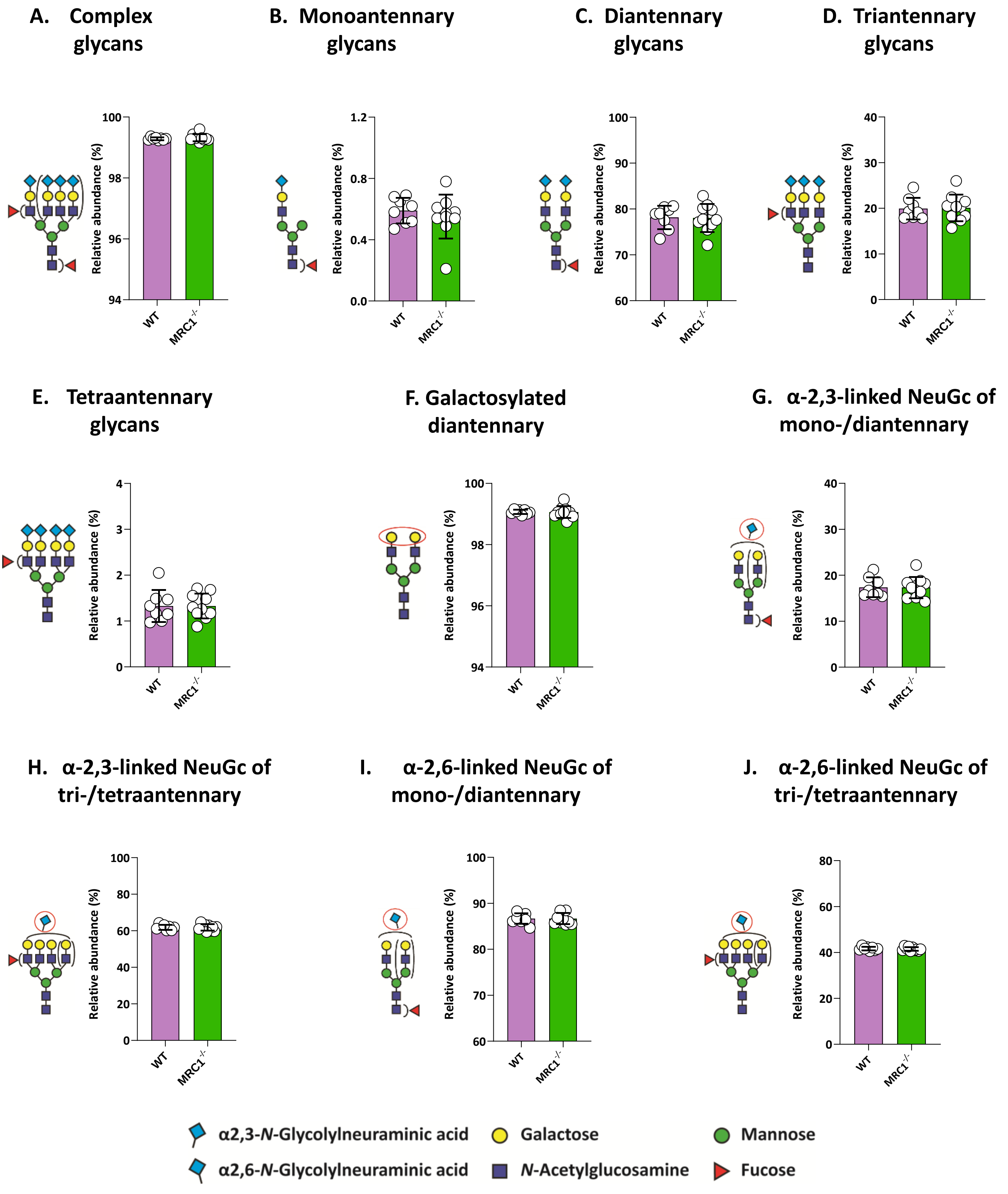

Supplementary Figure S5 – Glycosylation traits in MRC1 deficient mice. A) Complex glycans, B) Monoantennary glycans, C) Diantennary glycans, D) Triantennary glycans, E) Tetraantennary glycans, F) Galactosylated diantennary glycans, G) α2,3-linked *N*-Glycolylneuraminic acid on mono-/diantennary glycans, H) α2,3-linked *N*-Glycolylneuraminic acid on tri-/tetraantennary glycans, I) α2,6-linked *N*-Glycolylneuraminic acid on mono-/diantennary glycans, J) α2,6-linked *N*-Glycolylneuraminic acid of tri-/tetraantennary glycans. Data are shown as mean ± SD of WT (n=8) and MRC1$^{-/-}$ (n=10).

Supplementary Figure S6

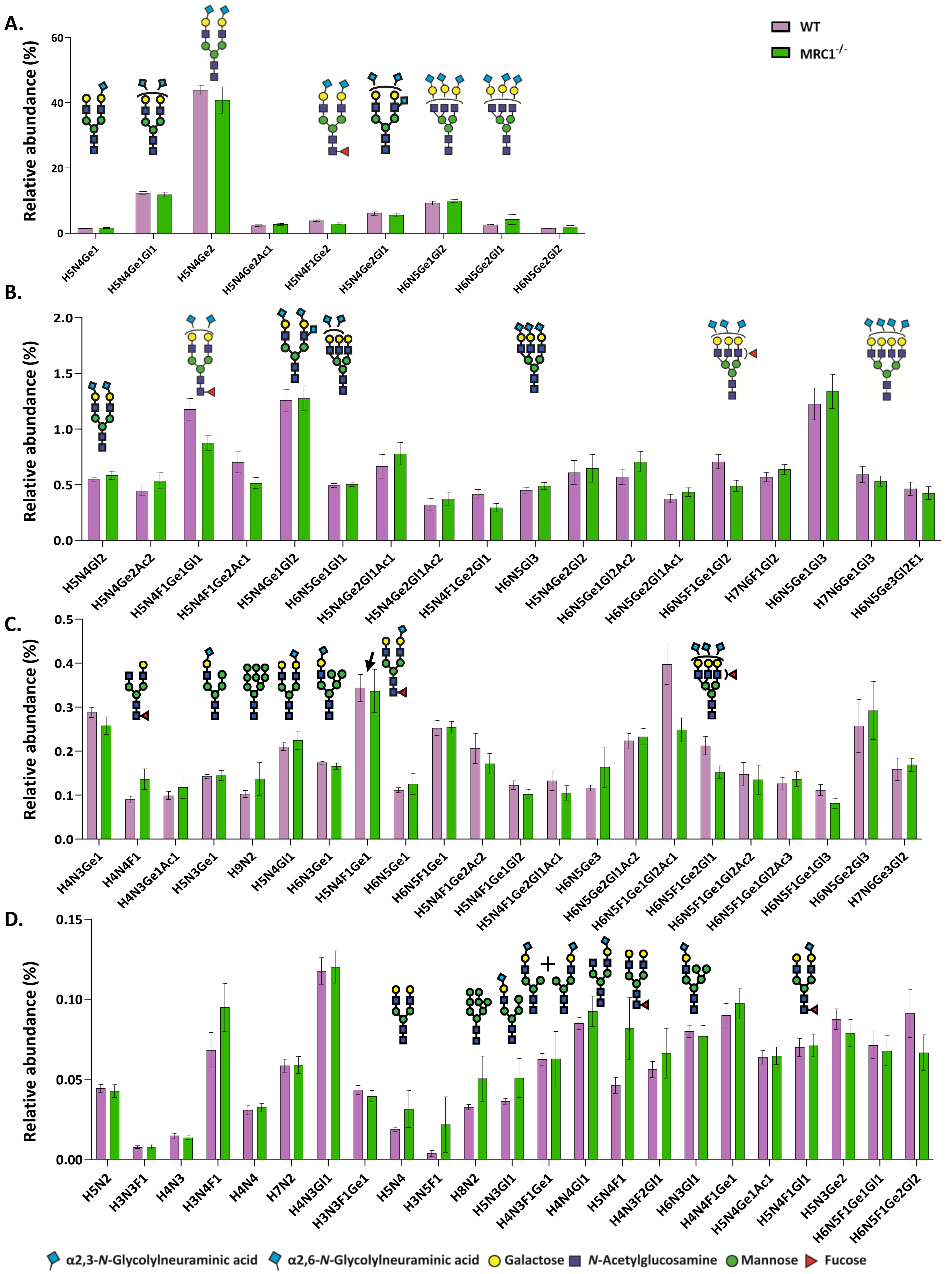

Supplementary Figure S6 – Glycan abundances in MRC1 deficient mice. Glycans are sorted by relative abundance into the different panels. A) >1.5%, B) 1.5% to 0.4%, C) 0.1% to 0.1%, D) <0.1%. In each panel, glycan abundances are depicted in ascending order of mass. Data are shown as mean ± SEM of WT (n=8) and MRC1$^{-/-}$ (n=10). H = hexose; N = *N*-Acetylhexosamine; F = fucose; E or L = *N*-Acetylneuraminic acid for α2,6- and α2,3-linked variants, respectively; Ge or Gl= *N*-Glycolylneuraminic acid for α2,6- and α2,3-linked variants, respectively; Ac = acetyl group.
